# The Antitumor Immunity and Tumor Responses of Chemotherapy with or without DC-CIK for Non-Small-Cell Lung Cancer in China: A Meta-Analysis of 28 Randomized Controlled Trials

**DOI:** 10.1155/2018/9081938

**Published:** 2018-12-13

**Authors:** Zheng Xiao, Cheng-qiong Wang, Ming-hua Zhou, Na-na Li, Yong-ping Sun, Yu-zhi Wang, Shi-yu Liu, Hong-song Yu, Cheng-wen Li, Xian-tao Zeng, Ling Chen, Xin-sheng Yao, Ji-hong Feng

**Affiliations:** ^1^Evidence-Based Medicine Center, MOE Virtual Research Center of Evidence-Based Medicine at Zunyi Medical College, Affiliated Hospital of Zunyi Medical College, Zunyi, 563000 Guizhou, China; ^2^Department of Respiratory Medicine (Center for Evidence-Based and Translational Medicine of Major Infectious Diseases), Affiliated Hospital of Zunyi Medical College, Zunyi, 563000 Guizhou, China; ^3^Teaching and Research Group of Evidence-based Medicine, Zhuhai Campus of Zunyi Medical College, Zhuhai, 519000 Guangdong, China; ^4^Department of immunology, Southwest Medical University, Luzhou, 646000 Sichuan, China; ^5^Department of Immunology, Special Key Laboratory of Gene Detection & Therapy of Guizhou Province, Zunyi Medical College, Zunyi, 563000 Guizhou, China; ^6^Center for Evidence-Based and Translational Medicine, Zhongnan Hospital of Wuhan University, Wuhan, 430071 Hubei, China; ^7^Department of Oncology, Affiliated Hospital of Zunyi Medical College, Zunyi, 563000 Guizhou, China

## Abstract

**Objective:**

DC-CIK therapy included DC-CIK cells and Ag-DC-CIK cells. To further confirm whether DC-CIK reconstructs the antitumor immunity and improves the tumor responses and reveals its optimal usage and combination with chemotherapy, we systematically reevaluated all the related studies.

**Materials and Methods:**

All studies about DC-CIK plus chemotherapy for NSCLC were collected from the published and ongoing database as CBM, CNKI, VIP, Wanfang, ISI, Embase, MEDLINE, CENTRAL, WHO-ICTRP, Chi-CTR, and US clinical trials (established on June 2017). We evaluated their methodological bias risk according to the Cochrane evaluation handbook of RCTs (5.1.0), extracted data following the predesigned data extraction form, and synthesized the data using meta-analysis.

**Results:**

We included 28 RCTs (phase IV) with 2242 patients, but most trials had unclear bias risk. The SMD and 95% CI of meta-analysis for CD3^+^ T cells, CD3^+^ CD4^+^ T cells, CD3^+^ CD8^+^ T cells, CD4^+^/CD8^+^ T cell ratio, CIK cells, NK cells, and Treg cells were as follows: 1.85 (1.39 to 2.31), 0.87 (0.65 to 1.10), 1.04 (0.58 to 1.50), 0.75 (0.27 to 1.22), 3.87 (2.48 to 5.25), 1.51 (0.99 to 2.03), and −2.31(−3.84 to −0.79). The RR and 95% CI of meta-analysis for ORR and DCR were as follows: 1.38 (1.24 to 1.54) and 1.27 (1.20 to 1.34). All differences were statistically significant between DC-CIK plus chemotherapy and chemotherapy alone. Subgroup analysis showed that only DC-CIK cells could increase the CD3^+^T cells, CD3^+^ CD4^+^T cells, CD3^+^ CD8^+^T cells, and CD4^+^/CD8^+^ T cell ratio. In treatment with one cycle or two cycles and combination with NP or GP, DC-CIK could increase the CD4^+^/CD8^+^ T cell ratio. All results had good stability.

**Conclusions:**

DC-CIK therapy can simultaneously improve the antitumor immunity and tumor responses. DC-CIK therapy, especially DC-CIK cells, can improve antitumor immunity through increasing the T lymphocyte subsets, CIK cell, and NK cells in peripheral blood. The one cycle to two cycles may be optimal cycle, and the NP or GP may be optimal combination.

## 1. Introduction

Non-small-cell lung cancer (NSCLC) remains the leading cause of cancer-related death worldwide [1–3]. Most clinically diagnosed patients undergo advanced local invasion and distant metastasis and therefore miss the opportunity of operative eradication. Hence, they are forced to accept the systemic chemotherapy. However, systemic chemotherapy damages host's immune function and weakens the antitumor immunity which result in poor tumor responses, survival, and quality of life (QOL) [4–6]. Therefore, how to repair and reconstruct antitumor immunity is the key to successful treatment. Dendritic cells (DC) are the most powerful antigen-presenting cells so far and play an important regulating role in host's immune response. DCs capture and process the tumor-associated antigens and then activate the antigen-specific cytotoxic T lymphocytes and induce the antitumor immune responses. Especially, dendritic cells pulsed with tumor-associated antigen(s) (Ag-DC cells) have stronger activity in mediating the antitumor immune responses than DC cells alone in vitro and in vivo [7–9]. Cytokine-induced killer cells (CD3^+^ CD56^+^ cells, CIK cells) were first described by Schmidt-Wolf et al. in 1991 [10], who also performed the first clinical trial with CIK cells in the treatment of cancer patients in 1999 [11]. CIK cells are nonmajor histocompatibility complex-restricted natural killer T lymphocytes and have stronger cytolytic activities against tumor than lymphokine-activated killer cells [12–16]. Coculture of DCs or Ag-DC cells and CIK cells results in considerable increase of antitumor immunity and shows stronger cytotoxic activity than single CIK treatment [17–19]. Therefore, DC cells or Ag-DC cells cocultured with CIK cells and formed DC-CIK therapy which are important cellular immunotherapy including the DC-CIK cells and Ag-DC-CIK cells.

DC-CIK therapy has been widely studied and applied in many kinds of malignant tumors [20–24]. Whether DC-CIK therapy repair and reconstruct antitumor immunity is the primary question to successful treatment. Antitumor immunity is expressed by indicators such as T lymphocyte subsets, natural killer cells (NK cells), and Th1 cytokines which are of great value for early judgement of clinical efficacy in DC-CIK treatment. Previous studies [25, 26] reported that CIK/DC-CIK therapy could significantly increase the proportion of CD3^+^ T cells, CD3^+^ CD4^+^ T cells, and the ratio of CD4^+^/CD8^+^ T cells and improve the tumor responses for NSCLC. However, this meta-analysis included the CIK and DC-CIK therapy, not focused on DC-CIK therapy. Lan et al. [27] further reported that the immunotherapy of DC-CIK cells significantly increased the proportion of CD3^+^ T cells, CD3^+^ CD4^+^ T cells, and the ratio of CD4^+^/CD8^+^ T cells and decreased the CD3^+^ CD8^+^ T cells in malignant tumors. This meta-analysis involved the rectal cancer, colorectal cancer, breast cancer, and NSCLC, not focused on NSCLC. Can DC-CIK therapy repair and reconstruct the antitumor immunity for NSCLC? In 2015, Hu et al. [28] reported that compared with chemotherapy, DC-CIK therapy could significantly increase the CD3^+^ T cells, CD3^+^ CD4^+^ T cells, and CD4^+^/CD8^+^ T cell ratio in NSCLC. The proportions of CD3^+^ CD8^+^ T cells were not statistically different between the two groups. However, Sun et al. [29] further reported that compared with platinum chemotherapy, DC-CIK therapy could significantly increase the proportions of CD3^+^ T cells, CD3^+^ CD4^+^ T cells, and the ratio of CD4^+^/CD8^+^ T cells in NSCLC. In 2016, Zhou et al. [30] reported that compared with chemotherapy, DC-CIK therapy could only increase the proportions of CD3^+^ T cells, natural killer cells (NK cells), and CIK cells. But the proportions of CD3^+^ CD4^+^ T cells, CD3^+^ CD8^+^ T cells, and CD25^+^ CD4^+^T cells (Treg cells) were not statistically different between the two groups. The results indicated that DC-CIK therapy might repair and reconstruct antitumor immunity for NSCLC through upregulating the T lymphocyte subsets and NK cells in peripheral blood. And there was controversy whether DC-CIK therapy improved the CD3^+^ CD8^+^ T cells. What usage and combinations with chemotherapy could improve the antitumor immunity remains unclear. In addition, DC-CIK cells and Ag-DC-CIK cells are different from each other. Therefore, current evidences [28–30] fail to answer whether DC-CIK cells or Ag-DC-CIK cells improve the antitumor immunity.

In clinical practice, there was outstanding diversity in different DC-CIK therapy and their usages and combinations with chemotherapy. All these might show different effects on clinical efficacy and then might be important factors in hindering the success of individualized immunotherapy. Up to now, many studies [31–33] had been published. Therefore, to further confirm whether DC-CIK therapy repairs and reconstructs the antitumor immunity, reveals its optimal usage and combination with chemotherapy, and provides the optimal evidence for individualized immunotherapy, we systematically reevaluated all the related studies.

## 2. Materials and Methods

This study was implemented according to the Preferred Reporting Items for Systematic Reviews and Meta-Analyses guidelines (PRISMA guidelines). Ethical approval was not required, as all materials of this study were published or unpublished studies.

### 2.1. Search Strategy

Two reviewers (Cheng-qiong Wang and Ming-hua Zhou) independently retrieved all the published studies in Chinese and English databases as Chinese Biomedical Literature (CBM), Chinese Scientific Journals Full-Text Database (CNKI), China National Knowledge Infrastructure Database (VIP) and Wanfang, Web of Science (ISI), Embase, MEDLINE, and Cochrane Central Register of Controlled Trials (CENTRAL) and further retrieved all the ongoing studies in WHO International Clinical Trials Registry Platform (WHO-ICTRP), Chinese Clinical Trial Registry (Chi-CTR), and US clinical trials (established on June 2017). All retrievals were implemented by using the Chinese and English MeSH and free word as “Dendritic cell and Cytokine Induced Killer Cells”, “Dendritic cells and Cytokine-Induced Killer Cell”, “Dendritic cell and Cytokine-Induced Killer Cell”, “Dendritic cells and Cytokine Induced Killer Cells”, “DCs CIK”, “DC CIK”, “DC Cik”, “DCs Cik”, “Lung Neoplasms”[Mesh], “Non-Small-Cell Lung” [Mesh], “Non small cell lung cancer”, “Non small cell lung cancers”, “Carcinoma, non-small cell lung cancer”, “Non-small cell lung cancers”, “NSCLC”, “Pulmonary Neoplasms”, “Lung Neoplasm”, “Pulmonary Neoplasm”, “Lung Cancer”, “Lung Cancers”, “Pulmonary Cancer”, “Pulmonary Cancers”, “Lung carcinoma” and “Pulmonary carcinoma”. Finally, we identified and evaluated all related systematic reviews (SRs) or meta-analysis and then selected all the studies meeting the inclusion criteria from their references.

### 2.2. Inclusion and Exclusion Criteria

Included studies must meet the following criteria. The disease was diagnosed as NSCLC using histopathological and cytological diagnostic criteria and TNM staging system [34]. There were no severe liver or kidney dysfunctions in any of the patients. Before being included, all patients did not receive the surgery, radiotherapy, CIK cells alone, traditional Chinese medicines, monoclonal antibody, or other cell therapies. DC-CIK therapy included DC-CIK cells and Ag-DC-CIK cells. The experimental groups were DC-CIK plus chemotherapy, and the control groups were chemotherapy alone without restrictions on the types. Main outcomes were antitumor immunity, and secondary outcomes were tumor responses. All studies were randomized controlled trials (RCTs). No restrictions were set on the follow-ups and types of research institutes.

Excluded studies must meet the following criteria: duplicates; patent, generic, abstracts, and reviews without specific data; in vitro or animal studies; studies about other tumors or nursing; studies with CIK cells or DC-CIK alone; studies with DC-CIK plus radiotherapy, Chinese herbs, targeted therapy, surgery, or other cytotherapy; studies with DC-CIK in two groups; nonrandomized controlled studies; unrelated SR or meta-analysis; studies without data of peripheral blood lymphocytes; and studies without being included in CBM.

### 2.3. Study Selection

Two reviewers (Shi-yu Liu and Na-na Li) independently selected the qualified studies in accordance with the predesigned inclusion and exclusion criteria. Any disagreements about selection were eliminated through discussing between themselves or with Zheng Xiao.

### 2.4. Bias Risk Assessment

Two reviewers (Yu-zhi Wang and Yong-ping Sun) evaluated the bias risk in accordance with the Cochrane evaluation handbook of RCTs (5.1.0) [35]. The bias parameters were the random sequence generation (selection bias), the allocation concealment (selection bias), the blinding of patients and researchers (performance bias), the blinding of outcome assessors (detection bias), the follow-up (attrition bias), the selective reporting (reporting bias), and the other bias (not comparable baseline). We judged each item on three levels (“yes” for a low risk of bias, “no” for a high risk of bias, and “unclear”). Then, we assessed the studies and categorized them into three levels: low risk of bias (low risk of bias for all key domains), high risk of bias (high risk of bias for one or more key domains), and unclear (unclear risk of bias for all key domains). Any disagreements of judgment about “high risk, low risk, or unclear” were resolved through discussing between themselves or with the third reviewer (Zheng Xiao).

### 2.5. Outcome Measures

We evaluated antitumor immunity using the peripheral blood lymphocytes that included T lymphocyte subsets and natural killer cells (NK cells). T lymphocyte subsets were measured by using the proportions of CD3^+^ T cells, CD3^+^ CD4^+^ T cells, CD3^+^ CD8^+^ T cells, CIK cells (CD3^+^ CD56^+^ cells), and regulatory T cells (CD25^+^ CD4^+^ T cells, Treg cells) and the ratio of CD4^+^/CD8^+^ T cells. All indicators were detected by using the flow cytometry (FCM) or other methods after treatment. Tumor responses were measured by using the objective response rate (ORR) and disease control rate (DCR). According to the World Health Organization (WHO) guidelines for solid tumor responses [36] or response evaluation criteria in solid tumors (RECIST) [37], indicators were complete response (CR), partial response (PR), no change (NC), progressive disease (PD), ORR being equal to CR plus PR, and DCR being equal to CR plus PR and NC.

### 2.6. Data Extraction

Two reviewers (Yu-zhi Wang and Hong-song Yu) independently extracted all the data in accordance with predesigned data extraction form, and all the data included the publishing time and author, the demographic characteristics, the sample sizes of experimental and control groups, DC-CIK types and usages, combinations with chemotherapy, evaluation methods and times, and main outcomes as the proportions of CD3^+^ T cells, CD3^+^ CD4^+^ T cells, CD3^+^ CD8^+^ T cells, CIK cells, regulatory T cells (Treg cells), and NK cells, and the ratio of CD4^+^/CD8^+^ T cells, secondary outcomes as ORR and DCR.

### 2.7. Statistical Analysis

Two reviewers (Cheng-qiong Wang and Ming-hua Zhou) implemented the meta-analysis using the Review Manager 5.3 (The Cochrane Collaboration, Oxford, UK). The relative risk (RR) and standardized mean difference (SMD) and 95% confidence intervals (CI) were used to describe the dichotomous and continuous variables, respectively. Statistical heterogeneity across trials was assessed by Pearson's chi-square test and *I* ^2^ test [38]. When substantial heterogeneity (*P* < 0.1, *I* ^2^ > 50%) was rejected, the fixed-effects model (FEM) was used to calculate the summary RR and the 95% CI. Otherwise, the data was calculated through a random-effects model (REM). To show the clinical heterogeneity and its influence on T lymphocyte subsets and further reveal the optimal usage and combination with chemotherapy of DC-CIK therapy, subgroup analysis was performed according to the DC-CIK types, treatment cycles, and different chemotherapy. Publication bias was evaluated by using the funnel plots when there were more than 10 included trials. Sensitivity analysis was performed through excluding the poor trials or over- or underestimated trials [39]. The trial was identified as poor trial that had at least one domain considered as high risk of bias. The trial was identified as over- or under-estimated trial, when results had statistical difference and positive effects on publication bias or heterogeneity.

## 3. Results

### 3.1. Search Results

**Figure 1:**
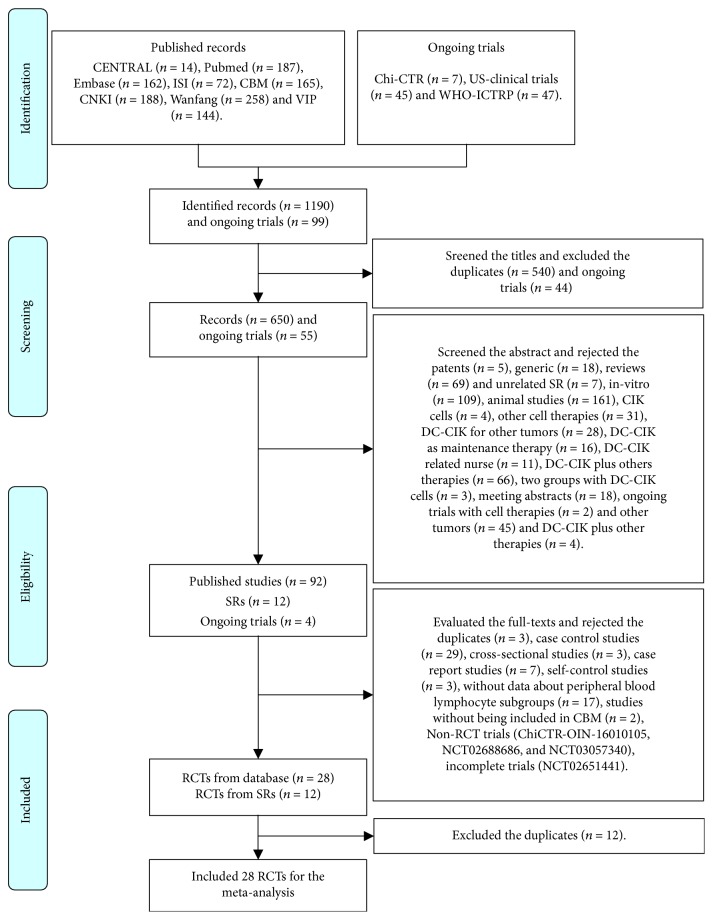
Articles retrieved and assessed for eligibility.

We retrieved 1190 published records and 99 ongoing trials through using the search strategies (Figure 1). We primarily excluded the duplicates and we included 650 records and 55 ongoing trials. We screened the abstracts and rejected the unrelated records et. al. and included 92 full-texts, 12 SRs [25–30, 40–45], and 4 ongoing trials. We further evaluated the full-text and SRs, rejected the unqualified studies, incomplete trials, and poor studies without being included in CBM, and we included 28 RCTs [31–33, 42, 46–69] from database and 12 RCTs [46–57] from SR or meta-analysis. Finally, we excluded the duplicates [46–57] and included 28 RCTs [31–33, 42, 46–69] for the meta-analysis.

### 3.2. Characteristics of Included Studies

In this meta-analysis, we included 28 RCTs [31–33, 42, 46–69] with 2242 NSCLC patients from China (Table 1). All patients were middle to late stage NSCLC without undergoing surgery. The males and females were 1256 cases and 792 cases with age range 18 to 83 years. DC-CIK plus chemotherapy and chemotherapy alone were 1127 cases and 1115 cases, respectively. In experimental groups, the patients underwent the DC-CIK cells in 22 RCTs [31, 33, 42, 46–50, 52, 54, 55, 57–65, 67, 68] and Ag-DC-CIK cells in 6 RCTs [32, 51, 53, 56, 66, 69], respectively. DC-CIK cells were mainly used by intravenous injection with 1–10 × 10^9^/times, 2–6 times/cycle, and 1–6 cycles after chemotherapy. The control groups underwent chemotherapy alone as vinorelbine and cisplatin (NP), docetaxel and cisplatin (DP), paclitaxel and cisplatin (TP), gemcitabine and cisplatin (GP), et al. All the trials evaluated antitumor immunity using the peripheral blood lymphocytes after treatment. The cells were mainly detected by using the flow cytometry (FCM). Twenty-four trials [31–33, 42, 46–52, 54–61, 63, 65, 66, 68, 69] with 1949 cases reported the tumor responses according to the WHO guidelines for solid tumor responses [36] or RECIST [37], respectively.

### 3.3. Methodological Bias Risk

**Figure 2 fig2:**
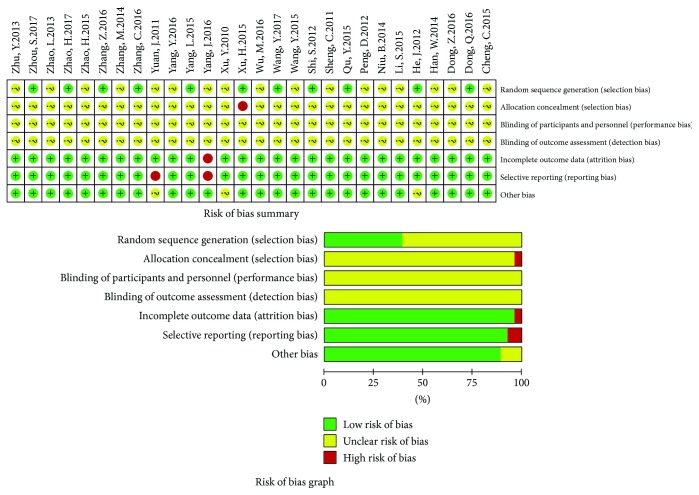
Methodological bias risk of included trials.

In 28 trials, only 11 trials reported the random sequence generation using the random number table [32, 33, 49, 51, 58–61, 63, 68, 69]. One trial [60] was allocation open, other trials did not provide the detailed information about the allocation concealment. None of the trials provided the detailed information about the blindings. One trial [66] had loss to follow-up. Two trials [48, 66] failed to completely report the tumor responses. Except for three trials [46, 48, 49], others were baseline comparability. Methodological bias risk was presented in Figure 2.

### 3.4. Peripheral Blood T Lymphocyte Subsets

**Figure 3 fig3:**
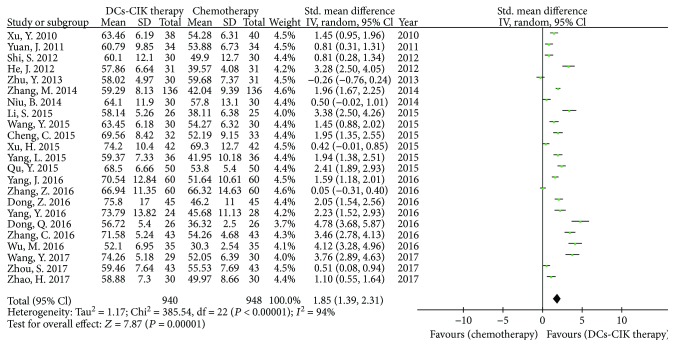
The analysis of CD3^+^ T cells between the two groups.

In 28 RCTs, 23 trials with 1888 cases reported the CD3^+^ T cells (Figure 3). Pearson's chi-square test and *I* ^2^ test showed that there was statistical heterogeneity among the trials (Chi^2^ = 385.54, *P* < 0.00001, *I* ^2^ = 94%). Therefore, the data was calculated by using a REM. The meta-analysis result showed that the proportions of CD3^+^ T cells had statistical difference between DC-CIK plus chemotherapy and chemotherapy alone [SMD = 1.85, 95% CI (1.39 to 2.31), *P* < 0.00001].

**Figure 4 fig4:**
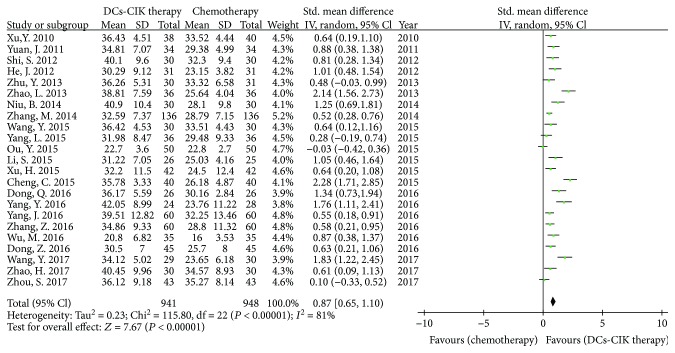
The analysis of CD4^+^ T cells between the two groups.

Twenty-three trials with 1889 cases reported the CD3^+^ CD4^+^ T cells (Figure 4). There was statistical heterogeneity among the trials (Chi^2^ = 115.80, *P* < 0.00001, *I* ^2^ = 81%). Therefore, the data was calculated by using a REM. The meta-analysis result showed that the CD3^+^ CD4^+^ T cells had statistical difference between the two groups [SMD = 0.87, 95% CI (0.65 to 1.10), *P* < 0.00001].

**Figure 5 fig5:**
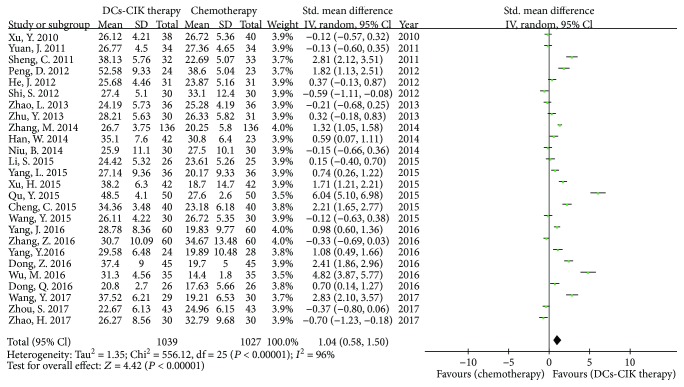
The analysis of CD8^+^ T cells between the two groups.

Twenty-six trials with 2066 cases reported the CD3^+^ CD8^+^ T cells (Figure 5). There was statistical heterogeneity among the trials (Chi^2^ = 556.12, *P* < 0.00001, *I* ^2^ = 96%). Therefore, the data was calculated by using a REM. The meta-analysis result showed that the CD3^+^ CD8^+^ T cells had statistical difference between the two groups [SMD = 1.04, 95% CI (0.58 to 1.50), *P* < 0.00001].

**Figure 6 fig6:**
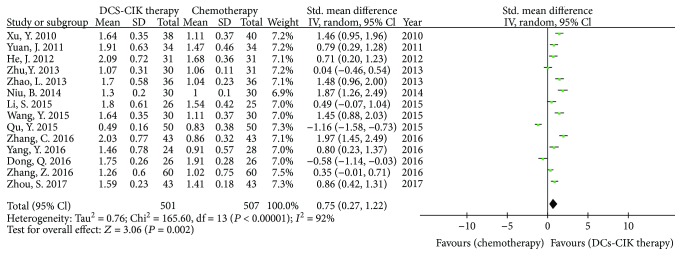
The analysis of CD4^+^/CD8^+^ T cells between the two groups.

Fifteen trials with 1068 cases reported the CD4^+^/CD8^+^ T cell ratio (Figure 6). There was statistical heterogeneity among the trials (Chi^2^ = 165.60, *P* < 0.00001, *I* ^2^ = 92%). Therefore, the data was calculated by using a REM. The meta-analysis result showed that the ratio of CD4^+^/CD8^+^ T cells had statistical difference between the two groups [SMD = 0.75, 95% CI (0.27 to 1.22), *P* = 0.002].

**Figure 7 fig7:**
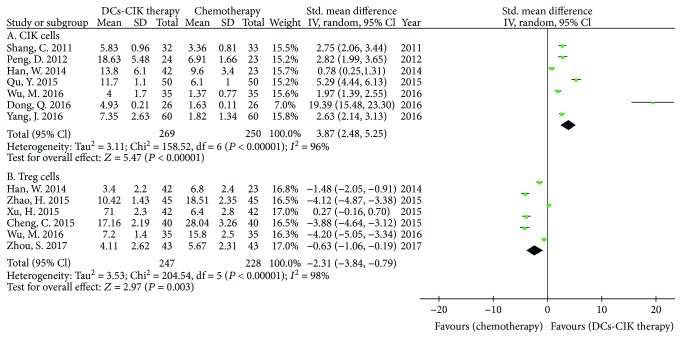
The analysis of CIK and Treg cells between the two groups.

Only 7 trials with 519 cases reported the CIK cells (Figure 7(a)). There was statistical heterogeneity among the trials (Chi^2^ = 158.52, *P* < 0.00001, *I* ^2^ = 96%). Therefore, the data was calculated by using a REM. The meta-analysis result showed that the CIK cells had statistical difference between the two groups [SMD = 3.87, 95% CI (2.48 to 5.25), *P* < 0.00001].

Only 6 trials with 475 cases reported the CD25^+^ CD4^+^ T cells (Treg cells) (Figure 7(b)). There was statistical heterogeneity among the trials (Chi^2^ = 204.54, *P* < 0.00001, *I* ^2^ = 98%). Therefore, the data was calculated by using a REM. The meta-analysis result showed that the Treg cells had statistical difference between the two groups [SMD = −2.31, 95% CI (−3.84 to −0.79), *P* = 0.003].

### 3.5. Natural Killer Cells (NK Cells)

**Figure 8 fig8:**
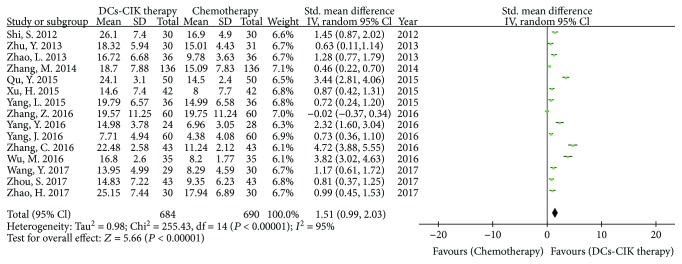
The analysis of NK cells between the two groups.

In 28 trials, 15 trials with 1374 cases reported the NK cells (Figure 8). There was statistical heterogeneity among the trials (Chi^2^ = 255.43, *P* < 0.00001, *I* ^2^ = 95%). Therefore, the data was calculated by a REM. The meta-analysis result showed that the NK cells had statistical difference between the two groups [SMD = 1.51, 95% CI (0.99 to 2.03), *P* < 0.00001].

### 3.6. Tumor Responses

**Figure 9 fig9:**
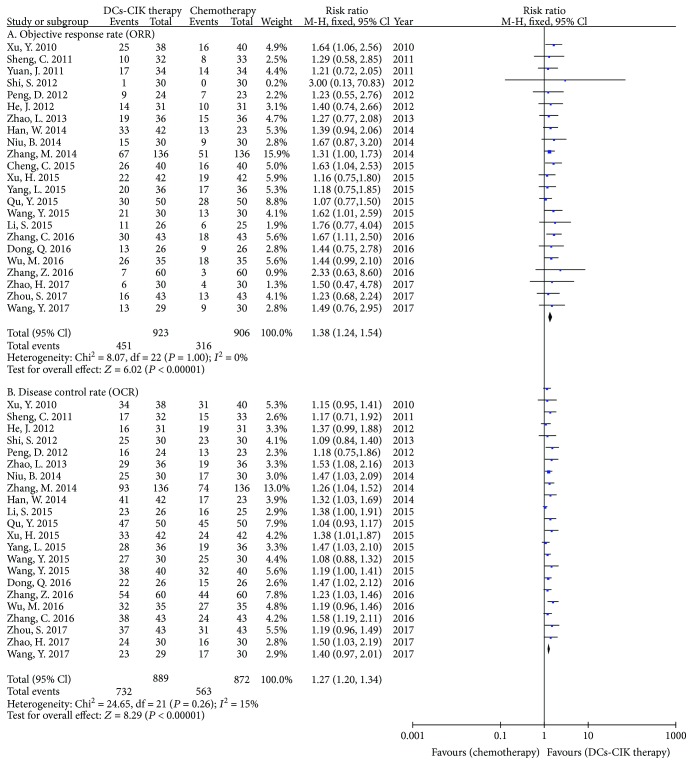
The analysis of tumor responses between the two groups.

According to the guidelines for solid tumor responses, tumor responses were evaluated by using the ORR and DCR. In 28 RCTs, 23 trials with 1829 cases reported the ORR. There was no statistical heterogeneity among the trials (Chi^2^ = 8.07, *P* = 1.00, *I* ^2^ = 0%). Therefore, the data were calculated by using a FEM. Compared with chemotherapy alone, the meta-analysis result showed that DC-CIK plus chemotherapy increased the ORR. The difference was statistically significant between DC-CIK plus chemotherapy and chemotherapy alone (RR = 1.38, 95% CI 1.24 to 1.54, *P* < 0.00001, Figure 9(a)). Twenty-two trials with 1761 cases reported the DCR. There was minimal heterogeneity among the trials (Chi^2^ = 24.65, *P* = 0.26, *I* ^2^ = 15%). Therefore, the data were calculated by using a FEM. The meta-analysis result showed that DC-CIK plus chemotherapy increased the DCR, and the difference was statistically significant between the two groups (RR = 1.27, 95% CI 1.20 to 1.34, *P* < 0.00001, Figure 9(b)).

### 3.7. Subgroup Analysis

To reveal the clinical heterogeneity and its influence on CD3^+^ T cells, CD3^+^ CD4^+^ T cells, CD3^+^ CD8^+^ T cells, and CD4^+^/CD8^+^ T cell ratio, subgroup analyses were performed according to the DC-CIK types, treatment cycles, and combinations with chemotherapy. Firstly, subgroup analyses showed that DC-CIK cells could increase the proportions of CD3^+^ T cells, CD3^+^ CD4^+^ T cells, CD3^+^ CD8^+^ T cells, and the ratio of CD4^+^/CD8^+^ T cells, but Ag-DC-CIK cells could only increase the CD3^+^ T cells and CD3^+^ CD4^+^ T cells (Table 2, Figure S1–4). Secondly, in treatment with one cycle or three cycles, DC-CIK therapy could increase the CD3^+^ T cells, CD3^+^ CD4^+^ T cells, and CD3^+^ CD8^+^ T cells. Treatment with one cycle to four cycles could all increase the proportions of CD3^+^ T cells and CD3^+^ CD4^+^ T cells. But only treatment with one cycle or two cycles could increase the ratio of CD4^+^/CD8^+^ T cells (Table 2, Figure S5–8). Thirdly, combinations with taxanes, NP, GP, or pemetrexed chemotherapy, DC-CIK could increase the CD3^+^ T cells and CD3^+^ CD4^+^ T cells. In combinations with taxanes, NP, or pemetrexed chemotherapy, DC-CIK could increase the CD8^+^ T cells. Only combinations with NP or GP, DC-CIK could increase the ratio of CD4^+^/CD8^+^ T cells (Table 2, Figure S9–12).

### 3.8. Publication Bias Analysis

**Figure 10:**
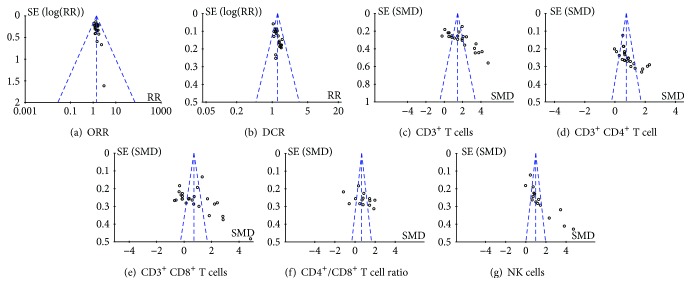
The analysis of publication bias.

The funnel plots were symmetrical in ORR (Figure 10(a)), and there was no publication bias in these trials. The funnel plots were significantly asymmetrical in DCR, CD3^+^ T cells, CD3^+^ CD4^+^ T cells, CD3^+^ CD8^+^ T cells, CD4^+^/CD8^+^ T cell ratio, and NK cells (Figures 10(b), 10(c), 10(d), 10(e), 10(f), and 10(g)), and there was publication bias in them. The results revealed that DCR was underestimated in one trial. The proportions of CD3^+^ T cells, CD3^+^ CD4^+^ T cells, CD3^+^ CD8^+^ T cells, and NK cells, and the CD4^+^/CD8^+^ T cell ratio were overestimated and underestimated, respectively.

### 3.9. Sensitivity Analysis

There were three poor trials [48, 60, 66] in this meta-analysis. One trial [60] had selection bias, one trial [66] had loss to follow-up, and two trials [48, 66] failed to completely report the tumor responses. There were poor trials in ORR, DCR, CD3^+^ T cells, CD3^+^ CD4^+^ T cells, CD3^+^ CD8^+^ T cells, NK cells, and CIK cells. Therefore, the sensitivity was evaluated through rejecting the poor trials. Before and after rejecting the poor trials, all results had good consistency (Table 3(a)). There was minimal heterogeneity and publication bias in DCR. There were statistical heterogeneity and publication bias in CD3^+^ T cells, CD3^+^ CD4^+^ T cells, CD3^+^ CD8^+^ T cells, CD4^+^/CD8^+^ T cell ratio, and NK cells. And there was only statistical heterogeneity in CIK cells and Treg cells. The DCR was underestimated and other indicators were, respectively, over- or underestimated, which might have positive effect on result's robustness. Therefore, the sensitivity was evaluated through rejecting the over- or underestimated trials. Before and after rejecting the over- or underestimated trials, all results had good consistency (Table 3(b)). In all, the meta-analysis results had good robustness.

## 4. Discussion

In this meta-analysis, we included 28 RCTs [31–33, 42, 46–69] with 2242 middle to late stage NSCLC patients without accepting surgery from China. Patients were 1311 males and 837 females between 18 and 85 years of age. DC-CIK therapy were Ag-DC-CIK cells and DC-CIK cells. The experimental groups underwent DC-CIK plus chemotherapy and the control groups underwent chemotherapy alone as DP, TP, GP, NP, et al. The DC-CIK cells were mainly used with 1–10 × 10^9^/times, 2–6 times/cycle, and 1–6 cycles through intravenous injection after chemotherapy. Antitumor immunity and tumor responses were, respectively, evaluated at 1–4 months after treatment.

Systematic chemotherapy significantly damage antitumor immunity, which is an important cause of poor tumor response and prognosis in patients with malignant tumors [70–72]. As an important cellular immunotherapy, the application of DC-CIK plus chemotherapy was clinically used in a wide range [20]. DC-CIK therapy were Ag-DC-CIK cells and DC-CIK cells. Previous meta-analysis [28–30] had failed to reveal whether DC-CIK cells or Ag-DC-CIK cells improve the antitumor immunity. And there was controversy whether DC-CIK therapy increase the CD3^+^ CD8^+^ T cells. What usage and combinations with chemotherapy could improve antitumor immunity remains unclear. In this meta-analysis, we included 28 trials with 2242 patients to evaluate the antitumor immunity. The meta-analysis results showed that DC-CIK therapy significantly increased the proportions of CD3^+^T cells, CD3^+^ CD4^+^ T cells, CD3^+^ CD8^+^ T cells, and the CD4^+^/CD8^+^ T cell ratio in peripheral blood. The included trials and sample sizes were sufficient for the analysis. The sensitivity analysis showed that the results had good robustness. But most trials had unclear bias risk. To compare with previous studies [28–30], this meta-analysis further confirmed that DC-CIK therapy significantly increased the proportions of T lymphocyte subgroups in peripheral blood. We found that DC-CIK therapy significantly increased the CD3^+^ CD8^+^ T cells. Related meta-analysis [73–75] had shown that DC-CIK could increase the CD3^+^ T cells, CD3^+^ CD4^+^ T cells, and the ratio of CD4^+^/CD8^+^ T cells in peripheral blood of patients with hepatocellular carcinoma (HCC) or gastric cancer. These studies provide indirect evidences for this meta-analysis's results. Therefore, we believed that DC-CIK therapy could significantly improve the antitumor immunity through upregulating the T lymphocytes. To show the clinical heterogeneity and its influence on T lymphocyte subsets and reveal the optimal usages and combination with chemotherapy of DC-CIK, subgroup analyses were performed according to the DC-CIK types, treatment cycles, and different chemotherapy. Subgroup analyses showed that DC-CIK cells significantly increase the T lymphocyte subsets. Ag-DC-CIK cells only increase the CD3^+^T cells and CD3^+^ CD4^+^ T cells. Therefore, whether Ag-DC-CIK cells improve the increase the T lymphocyte subsets still remains unclear. DC-CIK therapy was used with one cycle to six cycles. What was the optimal treatment cycle? The subgroup analysis showed that treatment with one cycle or two cycles, DC-CIK therapy could increase the CD4^+^/CD8^+^ T cell ratio. In addition, combinations with chemotherapies had complex and diverse characteristics. What was the optimal combinations? Further analysis revealed that only combinations with NP or GP, DC-CIK therapy could increase the CD4^+^/CD8^+^ T cell ratio. The results revealed that the DC-CIK types, treatment cycles, and combinations with chemotherapy were all important factors of clinical heterogeneity. To compare with previous studies [28–30], we further found that only DC-CIK cells could improve antitumor immunity through upregulating the T lymphocyte subsets in peripheral blood. Furthermore, only treatment with one cycle or two cycles, and combinations with NP or GP, DC-CIK therapy could improve the antitumor immunity through upregulating the CD4^+^/CD8^+^ T cell ratio.

CIK cells and NK cells are important effector cells in antitumor immunity. Can DC-CIK therapy increase the effector cells as CIK cells and NK cells? The meta-analysis results showed that DC-CIK therapy significantly increased the NK cells and CIK cells. Sensitivity analysis revealed that the results had good consistency. To compare with previous studies [28–30], this meta-analysis further revealed that DC-CIK could improve antitumor immunity though upregulating the CIK cells and NK cells. In addition, the regulatory T cells play an important role in immune tolerance and are important factors for downregulating the antitumor immunity. Therefore, how to break immune tolerance and downregulate the Treg cells also is an important way for antitumor immunotherapy. Six trials with 475 cases were included to reveal whether DC-CIK downregulate the Treg cells. The meta-analysis showed that DC-CIK therapy significantly decreased the proportions of Treg cells. But the included trials and sample sizes were insufficient for the analysis. Therefore, we believed that DC-CIK therapy might break immune tolerance through downregulating the Treg cells. In summary, DC-CIK therapy, especially DC-CIK cells, significantly improves antitumor immunity though upregulating the T lymphocyte subsets, NK cells, and CIK cells in peripheral blood. It also may improve antitumor immunity through downregulating the Treg cells and breaking the immune tolerance. Furthermore, only treatment with one cycle or two cycles, and combinations with NP or GP, DC-CIK therapy significantly improve the antitumor immunity. Therefore, we speculate that the one cycle to two cycles may be optimal cycle and the NP or GP might be optimal combinations. But whether Ag-DC-CIK cells improve the T lymphocytes still remains unclear. All these need to be revealed by new research.

Antitumor immunity only is secondary and short-term indicators for clinical efficacy. However, can DC-CIK therapy improve the tumor responses? In 28 trials, only 24 trials [31–33, 42, 46–52, 54–61, 63, 65, 66, 68, 69] with 1949 cases reported the tumor responses. The meta-analysis results revealed that DC-CIK plus chemotherapy increased the ORR and DCR. Sensitivity analysis showed that the results had good robustness. But, most trials had unclear bias risk. To compare with previous studies [28–30], this meta-analysis further confirmed that DC-CIK therapy improved the tumor responses. In vitro and vivo studies [16, 76, 77] had shown that CIK cells had highly efficient killing activity against a wide range of tumor cells. Other studies [19, 78, 79] also had confirmed that CIK cells co-cultured with dendritic cells could significantly enhance the antitumor activity. These studies provide basic evidences for this conclusion. We further confirmed that DC-CIK therapy could simultaneously improve the antitumor immunity and tumor responses. The results revealed that there was a correlation between antitumor immunity and tumor responses. We speculate that the antitumor immunity might play an important role in enhancing tumor responses. Therefore, detection of antitumor immunity after treatment might be of great value in predicting the tumor responses and prognosis. However, its potential laws still need to be revealed in further study.

There were some limitations in this study. Firstly, all the trials were published in China. Secondly, only 11 trials reported the random allocation method. All the trials did not provide the detailed information about the blindings. Thirdly, there were limited trials and sample sizes in Treg cells. Fourthly, cell number also had clinical heterogeneity. We did not perform subgroup analysis to reveal the influence of cell number on the T lymphocyte subsets, because most studies did not report it. Fifthly, in this meta-analysis, only 24 trials [31–33, 42, 46–52, 54–61, 63, 65, 66, 68, 69] with 1949 cases reported the tumor responses. All of these limitations might lead to an insufficient assessment for the antitumor immunity and tumor responses.

The available evidences indicate that DC-CIK therapy can simultaneously improve the antitumor immunity and tumor responses. DC-CIK therapy, especially DC-CIK cells can significantly improve antitumor immunity though up-regulating the T lymphocyte subsets, NK cells and CIK cells in peripheral blood. It also may improve antitumor immunity through down-regulating the Treg cells and breaking the immune tolerance. Therefore, we speculate that the one to two cycles may be optimal cycle and the NP or GP may be optimal combinations. Antitumor immunity might play an important role in enhancing the tumor responses. But whether Ag-DC-CIK cells improve the T lymphocytes remains unclear. But all these need to be further revealed by further larger scale clinical RCT or real-world studies. In this meta-analysis, we only reported the antitumor immunity and tumor responses. And we will further report the tumor responses, long-term survival, and safety in a new study. Finally, we hope that our results will provide valuable evidence for DC-CIK therapy, especially individualized immunotherapy.

## Figures and Tables

**Table 1:** Characteristics of included studies.

First author, year	NSCLC				Interventions			Criteria	Detecting	Time	O
	Stage	E/C	M/F	Years	Cells	Usage	C				
Xu, Y., 2010 [46]	III–IV	38/40	57/21	47–75	DC-CIK cells	CIK: 1.3–1.6^∗^10^9^/time (IV); DC (IC); 8–10 times/cycle, 2 cycles	NP	RECIST	FCM	4 w	O1, O2
Sheng, C., 2011 [47]	III-IV	32/33	37/28	35–65	DC-CIK cells	5^∗^10^9^/time, 4 times/cycle, 2 cycles (IV)	NP	WHO	FCM	2 w	O1, O2
Yuan, J., 2011 [48]	Advanced	34/34	42/26	Unclear	DC-CIK cells	Unclear, 4 times/cycle, 1 cycle (IV)	Chemo^∗^	WHO	Unclear	4 w	O1, O2
He, J., 2012 [49]	III–IV	31/31	Unclear	33–75	DC-CIK cells	Unclear, 2 times/cycle, 2 cycles (IV)	NP	WHO	Unclear	After	O1, O2
Peng, D., 2012 [50]	IIIa–IV	24/23	29/18	65–79	DC-CIK cells	DC: 1–10^∗^10^9^/time, CIK: 1–20^∗^^10^11/time, 2–3 times, 1 cycle (IV)	ALIMTA	RECIST	FCM	After	O1, O2
Shi, S. B., 2012 [51]	IIIb–IV	30/30	35/25	40–77	Ag-DC-CIK cells	DC: -, 4 times (SI); CIK: -, 5 times, 1 cycle (IV)	DP, GP	Unclear	FCM	After	O1, O2
Zhao, L., 2013[52]	IIIb–IV	36/36	Unclear	60–80	DC-CIK cells	Unclear, 3 times/cycle, 2 cycles (IV)	DP	RECIST	FCM	After	O1, O2
Zhu, Y., 2013 [53]	IIIb–IV	30/31	32/29	44–72	Ag-DC-CIK cells	Unclear, 4 times/cycle, 1cycle (IV)	TP	No	FCM	After	O1
Han, W., 2014 [54]	Advanced	42/23	45/20	Unclear	DC-CIK cells	5^∗^10^9^/time, 4 times/cycle, 4 cycles (IV)	DP	WHO	Unclear	After	O1, O2
Niu, B., 2014 [55]	III–IV	30/30	Unclear	32–77	DC-CIK cells	Unclear, 3 times/cycle, 1 cycle (IV)	GP	RECIST	Unclear	After	O1, O2
Zhang, M., 2014 [56]	IIIa–IV	136/136	139/133	18–75	Ag-DC-CIK cells	>1^∗^10^10^/cycle, 4 times/cycle, 1 cycle (IV)	TP, DP	RECIST	FCM	After	O1, O2
Cheng, C., 2015 [57]	Unclear	40/40	54/26	30–73	DC-CIK cells	Unclear, 8 times/cycle, 3 cycles (IV)	TP	WHO	FCM	After	O1, O2
Li, S., 2015[42]	III–IV	26/25	25/26	70–81	DC-CIK cells	Unclear, 2 times/cycle, 2–6cycles (IV)	GP	RECIST	FCM	1 w	O1, O2
Qu, Y., 2015 [58]	IIIb–IV	50/50	62/38	36–77	DC-CIK cells	4–5^∗^10^9^/cycle, 4 times/cycle, 6 cycles (IV)	TP	RECIST	FCM	1 w	O1, O2
Wang, Y., 2015 [59]	Advanced	30/30	35/25	40–65	DC-CIK cells	Unclear, 8–10 times/cycle, 2 cycles (IV)	NP	Unclear	Unclear	4 w	O1, O2
Xu, H., 2015 [60]	III–IV	42/42	56/28	54–79	DC-CIK cells	DC: >1^∗^10^7^/time, 4 times, CIK: >1^∗^10^9^/time, 2 times, 2 cycles	TP	RECIST	FCM	After	O1, O2
Yang, L., 2015 [61]	IIIa–IV	36/36	47/25	38–77	DC-CIK cells	>1^∗^10^10^/time, 4 times/cycle, 1 cycle (IV)	TN	RECIST	FCM	2 w	O1, O2
Zhao, H., 2015[62]	II–IIIb	45/45	66/24	50–79	DC-CIK cells	5^∗^10^9^/time, 5 times/cycle, 4 cycles (IV)	PP	No	FCM	After	O1
Dong, Q., 2016 [63]	III–IV	26/26	28/24	52–77	DC-CIK cells	>1^∗^10^10^/cycle, 5 times/cycle, 4 cycles (IV)	PP	RECIST	FCM	1 w	O1, O2
Dong, Z., 2016[64]	Advanced	45/45	46/44	43–80	DC-CIK cells	1.0^∗^10^9^/time, 24 times/cycle, 1 cycle	NP	No	Unclear	After	O1
Wu, M., 2016 [65]	IIIb–IV	35/35	52/18	Unclear	DC-CIK cells	DC: 1^∗^10^7^/time (SI), CIK: 4^∗^10^9^/time (IV), 5 times, 4 cycles	TP	WHO	FCM	4 w	O1, O2
Yang, J., 2016[66]	IIIb–IV	60/60	54/66	Unclear	Ag-DC-CIK cells	Unclear, 1 time/cycle, 2 cycles (IV)	PP	RECIST	FCM	After	O1, O2
Yang, Y., 2016 [67]	Advanced	24/28	29/23	65–75	DC-CIK cells	>1.0^∗^10^9^/cycle, 6 times/cycle, 1 cycle	TP	RECIST	Unclear	After	O1
Zhang, C., 2016 [68]	IIb–IIIb	43/43	51/35	40–83	DC-CIK cells	5^∗^10^9^/time, 4 times/cycle, 2–6 cycles (IV)	GP	Unclear	Unclear	After	O1, O2
Zhang, Z., 2016[69]	IIIb–IV	60/60	104/16	29–82	Ag-DC-CIK cells	DC: -, 2 times, CIK: >1^∗^10^8^/time, 3 times/cycle, unclear (IV)	Chemo^∗^	RECIST	FCM	After	O1, O2
Wang, Y., 2017 [31]	IIIa–IV	29/30	36/23	35–76	DC-CIK cells	1.0^∗^10^9^/time, 8 times/cycle, 3 cycles (IV)	NP	Unclear	FCM	After	O1, O2
Zhao, H., 2017 [32]	IIIb–IV	30/30	37/23	Unclear	Ag-DC-CIK cells	DC: -, 1 time (SI); CIK: -, 1 time (IV), 2 cycles	GP	RECIST	FCM	After	O1, O2
Zhou, S., 2017 [33]	II–IV	43/43	58/28	32–70	DC-CIK cells	Unclear, 5 times/cycle, 1 cycle (IV)	GP	RECIST	FCM	After	O1, O2
											

Note: NSCLC: non-small-cell lung cancer; E: experimental group (DC-CIK plus chemotherapy); C: control group (chemotherapy); chemo^∗^: chemotherapy; DP: docetaxel and cisplatin; TP: paclitaxel and cisplatin; TN: paclitaxel and nedaplatin; GP: gemcitabine and cisplatin; GP^∗^: gemcitabine and platinum; NP: vinorelbine and cisplatin; PP: pemetrexed and cisplatin; ALIMTA: pemetrexed disodium for injection; GO^∗^: gemcitabine and oxaliplatin; EP: etoposide and cisplatin; IV: intravenous injection; SI: subcutaneous injection lymph node-rich region; WHO: World Health Organization guidelines for solid tumor responses; RECIST: response evaluation criteria in solid tumors; FCM: flow cytometry; Outcomes: O1: cellular immunity; O2: tumor responses.

**Table 2 tab2:** Subgroup analysis results of peripheral blood T lymphocytes.

Subgroups	SM	CD3^+^ T cells				CD3^+^ CD4^+^ T cells				CD3^+^ CD8^+^ T cells				CD4^+^/CD8^+^ T cell ratio			
		Trials	Cases	SMD(95% CI)	I^2^	Trials	Cases	SMD(95% CI)	I^2^	Trials	Cases	SMD(95% CI)	I^2^	Trials	Cases	SMD(95% CI)	I^2^
Totality	REM	23	1888	1.85 [1.39, 2.31]	94%	23	1889	0.87 [0.65, 1.10]	81%	26	2066	1.04 [0.58, 1.50]	96%	14	1008	0.75 [0.27, 1.22]	92%
*(a) Subgroup analysis via DC-CIK types (Figure S1–4)*																	
DC-CIK cells	REM	17	1195	2.21 [1.64, 2.78]	94%	17	1196	0.99 [0.68, 1.31]	85%	20	1373	1.32 [0.72, 1.91]	96%	12	827	0.84 [0.28, 1.40]	93%
Ag-DC-CIK cells	REM	6	693	0.88 [0.11, 1.65]	95%	6	693	0.57 [0.41, 0.72]	0%	6	693	0.18 [−0.56, 0.92]	95%	2	181	0.25 [−0.05, 0.54]	0%
*(b) Subgroup analysis via treatment cycles (Figure S5–8)*																	
One cycle	REM	9	821	1.16 [0.59, 1.74]	92%	9	821	0.70 [0.42, 0.98]	70%	10	868	0.64 [0.05, 1.23]	94%	5	327	0.86 [0.33, 1.38]	81%
Two cycles	REM	6	464	1.51 [0.88, 2.13]	89%	7	536	0.87 [0.50, 1.23]	75%	8	601	0.57 [−0.12, 1.27]	94%	4	272	1.27 [0.90, 1.65]	51%
Three cycles	REM	2	124	2.83 [1.05, 4.60]	91%	2	139	2.07 [1.63, 2.51]	9%	2	139	2.47 [1.87, 3.08]	43%	No	No	No	No
Four cycles	REM	2	122	4.36 [3.69, 5.03]	0%	2	122	1.07 [0.62, 1.52]	25%	3	187	2.00 [−0.11, 4.11]	97%	1	52	−0.58 [−1.14, −0.03]	No
Six cycles	REM	1	100	2.41 [1.89, 2.93]	No	1	100	−0.03 [−0.42, 0.36]	No	1	100	6.04 [5.10, 6.98]	No	1	100	−1.16 [−1.58, −0.73]	No
Two to six cycles	REM	2	137	3.43 [2.89, 3.96]	0%	1	51	1.05 [0.46, 1.64]	No	1	51	0.15 [−0.40, 0.70]	No	2	137	1.23 [−0.22, 2.68]	93%
Unclear	REM	1	120	0.05 [−0.31, 0.40]	No	1	120	0.58 [0.21, 0.95]	No	1	120	−0.33 [−0.69, 0.03]	No	1	120	0.35 [−0.01, 0.71]	No
*(c) Subgroup analysis via combination with chemotherapy (Figure S9–12)*																	
Taxanes^∗^	REM	7	504	1.80 [0.81, 2.79]	95%	8	591	1.03 [0.44, 1.62]	91%	9	656	1.87 [0.86, 2.88]	97%	4	285	0.28 [−0.89, 1.46]	95%
NP	REM	5	349	2.34 [1.54, 3.15]	88%	5	349	0.92 [0.52, 1.32]	68%	6	414	1.34 [0.22, 2.47]	96%	3	200	1.20 [0.71, 1.69]	62%
GP	REM	5	343	1.75 [0.58, 2.93]	95%	4	257	0.73 [0.19, 1.26]	77%	4	257	−0.28 [−0.61, 0.05]	44%	4	283	1.29 [0.58, 2.00]	86%
Pemetrexed^∗^	REM	2	172	3.14 [0.03, 6.26]	96%	2	172	0.90 [0.14, 1.67]	79%	3	219	1.12 [0.57, 1.68]	69%	1	52	−0.58 [−1.14, −0.03]	
Systemic^∗^	REM	4	520	0.91 [−0.05, 1.87]	96%	4	520	0.61 [0.44, 0.79]	0%	4	520	0.08 [−0.92, 1.08]	96%	2	188	0.54 [0.11, 0.96]	49%

Note: REM: random-effects model; SMD: standardized mean difference; SM: statistical method; taxanes^∗^: taxane chemotherapy as paclitaxel and cisplatin (TP), paclitaxel and nedaplatin (TN), and docetaxel and cisplatin (DP); NP: navebine and cisplatin; GP: gemcitabine and cisplatin; pemetrexed^∗^: pemetrexed chemotherapy including pemetrexed and cisplatin and pemetrexed alone; systemic^∗^: systemic chemotherapy including GP, TP, DP, et al.

**Table:** (a) Sensitivity analysis through rejecting the poor trials.

Indicators	Trials	SM	Effect estimate SMD (95% CI)	*I* ^2^	Rejected trials^∗^	Trials	SM	Effect estimate SMD (95% CI)	*I* ^2^
ORR	23	FEM	1.38 [1.24, 1.54]	0%	Poor^∗^ [48, 60]	21	FEM	1.41 [1.26, 1.57]	0%
DCR	22	FEM	1.27 [1.20, 1.34]	15%	Poor^∗^ [60]	21	FEM	1.27 [1.20, 1.34]	16%
CD3^+^ T cells	23	REM	1.85 [1.39, 2.31]	94%	Poor^∗^ [60, 66]	21	REM	1.93 [1.43, 2.44]	94%
CD4^+^ T cells	23	REM	0.87 [0.65, 1.10]	81%	Poor^∗^ [60, 66]	21	REM	0.91 [0.66, 1.15]	83%
CD8^+^ T cells	26	REM	1.04 [0.58, 1.50]	96%	Poor^∗^ [60, 66]	24	REM	1.02 [0.52, 1.51]	96%
NK cells	15	REM	1.51 [0.99, 2.03]	95%	Poor^∗^ [60, 66]	13	REM	1.63 [1.01, 2.25]	95%
CIK cells	7	REM	3.87 [2.48, 5.25]	96%	Poor^∗^ [66]	6	REM	4.33 [2.56, 6.11]	97%

**Table:** (b) Sensitivity analysis through rejecting the over- or underestimated trials.

Indicators	Trials	SM	Effect estimate SMD (95% CI)	I^2^	Rejected trials^∗^	Trials	SM	Effect estimate SMD (95% CI)	I^2^
DCR	22	FEM	1.27 [1.20, 1.34]	15%	Over^∗^ [52, 68]	20	FEM	1.25 [1.18, 1.32]	0%
CD3^+^ T cells	23	REM	1.85 [1.39, 2.31]	94%	Over^∗^ [31, 32, 42, 46, 49, 56–59, 61, 63–68]; under^∗^ [53]	6	FEM	0.45 [0.27, 0.63]	43%
CD4^+^ T cells	23	REM	0.87 [0.65, 1.10]	81%	Over^∗^ [31, 52, 57, 67]	12	FEM	0.47 [0.35, 0.58]	16%
CD8^+^ T cells	26	REM	1.04 [0.58, 1.50]	96%	Over^∗^ [31, 47, 50, 56–58, 60, 64–67]; under^∗^ [32, 33, 51, 52, 69]	10	FEM	0.22 [0.06, 0.38]	50%
CD4^+^/CD8^+^T cell ratio	15	REM	0.74 [0.30, 1.19]	92%	Over^∗^ [46, 52, 55, 59, 68]; under^∗^ [58, 63]	8	FEM	0.56 [0.38, 0.74]	34%
NK cells	15	REM	1.51 [0.99, 2.03]	95%	Over^∗^ [51, 58, 65, 67, 68]; under^∗^ [69]	9	FEM	0.74 [0.60, 0.87]	39%
CIK cells	7	REM	3.87 [2.48, 5.25]	96%	Over^∗^ [58, 63]; under^∗^ [54]	4	FEM	2.50 [2.19, 2.80]	35%
Treg cells	6	REM	−2.31 [−3.84, −0.79]	98%	Over^∗^ [57, 62, 65]; under^∗^ [60]	2	REM	−1.03 [−1.87, −0.20]	82%
									

Note: RR: risk ratios; SMD: standardized mean difference; FEM: fixed-effects model; REM: random-effects model; CI: confidence interval; NK cells: natural killer cells; CIK cells: cytokine-induced killer cells; Treg cells: regulatory T cells; poor^∗^: poor trials that had at least one domain considered as high risk of bias; over^∗^ or under^∗^: over- or underestimated trials when the results had statistical difference and positive effects on publication bias or heterogeneity.

## Data Availability

Readers can access the data supporting the conclusions of the study from Figures 1 [Fig fig2] [Fig fig3] [Fig fig4] [Fig fig5] [Fig fig6] [Fig fig7] [Fig fig8] [Fig fig9]–10 and Tables 1 [Table tab2]–3. And other data can be accessed in the Optional Supplementary Materials including checklist and Appendix 2 (Figure S1–12. Subgroup analysis). In addition, if there is any need, please email us directly (zy426f@163.com).

## Supplementary Materials

Supplementary Materials Appendix 1: PRISMA checklist. Appendix 2: subgroup analysis (Figure S1–12). Figure S1: subgroup analysis of CD3^+^ T cell via different DC-CIK cells. Figure S2: subgroup analysis of CD4^+^ T cell via different DC-CIK cells. Figure S3: subgroup analysis of CD8^+^ T cell via different DC-CIK cells. Figure S4: subgroup analysis of CD4^+^/CD8^+^ T cell via different DC-CIK cells. Figure S5: subgroup analysis of CD3^+^ T cell via different cycles. Figure S6: subgroup analysis of CD4^+^ T cell via different cycles. Figure S7: subgroup analysis of CD8^+^ T cell via different cycles. Figure S8: subgroup analysis of CD4^+^/CD8^+^ T cell via different cycles. Figure S9: subgroup analysis of CD3^+^ T cell via different chemotherapy. Figure S10: subgroup analysis of CD4^+^ T cell via different chemotherapy. Figure S11: subgroup analysis of CD8^+^ T cell via different chemotherapy. Figure S12: subgroup analysis of CD4^+^/CD8^+^ T cell via different chemotherapy.
