# Supplementary material for: The Antitumor Immunity and Tumor Responses of Chemotherapy with or without DC-CIK for Non-Small-Cell Lung Cancer in China: A Meta-Analysis of 28 Randomized Controlled Trials
Source: J Immunol Res. 2018 Dec 13;2018:9081938. doi: 10.1155/2018/9081938 (PMC6311820; doi:10.1155/2018/9081938)
Supplement: Supplementary Materials — Appendix 1: PRISMA checklist. Appendix 2: subgroup analysis (Figure S1–12). Figure S1: subgroup analysis of CD3+ T cell via different DC-CIK cells. Figure S2: subgroup analysis of CD4+ T cell via different DC-CIK cells. Figure S3: subgroup analysis of CD8+ T cell via different DC-CIK cells. Figure S4: subgroup analysis of CD4+/CD8+ T cell via different DC-CIK cells. Figure S5: subgroup analysis of CD3+ T cell via different cycles. Figure S6: subgroup analysis of CD4+ T cell via different cycles. Figure S7: subgroup analysis of CD8+ T cell via different cycles. Figure S8: subgroup analysis of CD4+/CD8+ T cell via different cycles. Figure S9: subgroup analysis of CD3+ T cell via different chemotherapy. Figure S10: subgroup analysis of CD4+ T cell via different chemotherapy. Figure S11: subgroup analysis of CD8+ T cell via different chemotherapy. Figure S12: subgroup analysis of CD4+/CD8+ T cell via different chemotherapy. [file 9081938.f1.pdf]

Section/topic	#	Checklist item	Reported on page #
TITLE: **Can DCs-CIK plus chemotherapy improve antitumor immunity in non-small cell lung cancer: A meta-analysis of 30 randomized controlled trials**			
Title	1	Identify the report as a systematic review, meta-analysis, or both.	1
**ABSTRACT**			
Structured summary	2	Provide a structured summary including, as applicable: background; objectives; data sources; study eligibility criteria, participants, and interventions; study appraisal and synthesis methods; results; limitations; conclusions and implications of key findings; systematic review registration number.	1-2
**INTRODUCTION**			
Rationale	3	Describe the rationale for the review in the context of what is already known.	3-4
Objectives	4	Provide an explicit statement of questions being addressed with reference to participants, interventions, comparisons, outcomes, and study design (PICOS).	3-4
**METHODS**			
Protocol and registration	5	Indicate if a review protocol exists, if and where it can be accessed (e.g., Web address), and, if available, provide registration information including registration number.	No
Eligibility criteria	6	Specify study characteristics (e.g., PICOS, length of follow-up) and report characteristics (e.g., years considered, language, publication status) used as criteria for eligibility, giving rationale.	4-5
Information sources	7	Describe all information sources (e.g., databases with dates of coverage, contact with study authors to identify additional studies) in the search and date last searched.	4
Search	8	Present full electronic search strategy for at least one database, including any limits used, such that it could be repeated.	4
Study selection	9	State the process for selecting studies (i.e., screening, eligibility, included in systematic review, and, if applicable,	5

		included in the meta-analysis).	
Data collection process	10	Describe method of data extraction from reports (e.g., piloted forms, independently, in duplicate) and any processes for obtaining and confirming data from investigators.	6
Data items	11	List and define all variables for which data were sought (e.g., PICOS, funding sources) and any assumptions and simplifications made.	6
Risk of bias in individual studies	12	Describe methods used for assessing risk of bias of individual studies (including specification of whether this was done at the study or outcome level), and how this information is to be used in any data synthesis.	5
Summary measures	13	State the principal summary measures (e.g., risk ratio, difference in means).	6
Synthesis of results	14	Describe the methods of handling data and combining results of studies, if done, including measures of consistency (e.g., I^2) for each meta-analysis.	6

Section/topic	#	Checklist item	Reported on page #
Risk of bias across studies	15	Specify any assessment of risk of bias that may affect the cumulative evidence (e.g., publication bias, selective reporting within studies).	5
Additional analyses	16	Describe methods of additional analyses (e.g., sensitivity or subgroup analyses, meta-regression), if done, indicating which were pre-specified.	6
**RESULTS**			
Study selection	17	Give numbers of studies screened, assessed for eligibility, and included in the review, with reasons for exclusions at each stage, ideally with a flow diagram.	6-7

Study characteristics	18	For each study, present characteristics for which data were extracted (e.g., study size, PICOS, follow-up period) and provide the citations.	7-9
Risk of bias within studies	19	Present data on risk of bias of each study and, if available, any outcome level assessment (see item 12).	10
Results of individual studies	20	For all outcomes considered (benefits or harms), present, for each study: (a) simple summary data for each intervention group (b) effect estimates and confidence intervals, ideally with a forest plot.	6
Synthesis of results	21	Present results of each meta-analysis done, including confidence intervals and measures of consistency.	10-13
Risk of bias across studies	22	Present results of any assessment of risk of bias across studies (see Item 15).	10-13
Additional analysis	23	Give results of additional analyses, if done (e.g., sensitivity or subgroup analyses, meta-regression [see Item 16]).	14-18
**DISCUSSION**			
Summary of evidence	24	Summarize the main findings including the strength of evidence for each main outcome; consider their relevance to key groups (e.g., healthcare providers, users, and policy makers).	19-20
Limitations	25	Discuss limitations at study and outcome level (e.g., risk of bias), and at review-level (e.g., incomplete retrieval of identified research, reporting bias).	20-21
Conclusions	26	Provide a general interpretation of the results in the context of other evidence, and implications for future research.	21
**FUNDING**			
Funding	27	Describe sources of funding for the systematic review and other support (e.g., supply of data); role of funders for the systematic review.	21

From: Moher D, Liberati A, Tetzlaff J, Altman DG, The PRISMA Group (2009). Preferred Reporting Items for Systematic Reviews and Meta-Analyses: The PRISMA Statement. PLoS Med 6(7): e1000097. doi:10.1371/journal.pmed1000097

For more information, visit: **www.prisma-statement.org**.

Appendix 2

Figure S1-12. Subgroups analysis

Study or Subgroup	DCs-CIK Mean	DCs-CIK SD	DCs-CIK Total	Chemotherapy Mean	Chemotherapy SD	Chemotherapy Total	Weight	Std. Mean Difference IV, Random, 95% CI	Year
**DCs-CIK cells**									
Xu,Y. 2010	63.46	6.19	38	54.28	6.31	40	4.1%	1.45 [0.95, 1.96]	2010
Yuan,J.2011	60.79	9.85	34	53.88	6.73	34	4.1%	0.81 [0.31, 1.31]	2011
He,J.2012	57.86	6.64	31	39.57	4.08	31	3.8%	3.28 [2.50, 4.05]	2012
Niu,B.2014	64.1	11.9	30	57.8	13.1	30	4.1%	0.50 [-0.02, 1.01]	2014
Sui,C.2014	57.56	6.64	20	38.67	5.63	20	3.7%	3.01 [2.08, 3.94]	2014
Xu,H.2015	74.2	10.4	42	69.3	12.7	42	4.2%	0.42 [-0.01, 0.85]	2015
Wang,Y.2015	63.45	6.18	30	54.27	6.32	30	4.1%	1.45 [0.88, 2.02]	2015
Li,S.2015	58.14	5.26	26	38.11	6.38	25	3.7%	3.38 [2.50, 4.26]	2015
Cheng,C.2015	69.56	8.42	32	52.19	9.15	33	4.0%	1.95 [1.35, 2.55]	2015
Qu, Y. 2015	68.5	6.66	50	53.8	5.4	50	4.1%	2.41 [1.89, 2.93]	2015
Yang, L.2015	59.37	7.33	36	41.95	10.18	36	4.1%	1.94 [1.38, 2.51]	2015
Zhang,C.2016	71.58	5.24	43	54.26	4.68	43	4.0%	3.46 [2.78, 4.13]	2016
Wang, L.2016	57.88	6.66	30	39.21	4.31	30	3.8%	3.29 [2.49, 4.08]	2016
Wu, M.2016	52.1	6.95	35	30.3	2.54	35	3.8%	4.12 [3.28, 4.96]	2016
Dong, Z.2016	75.8	17	45	46.2	11	45	4.1%	2.05 [1.54, 2.56]	2016
Yang, Y.2016	73.79	13.82	24	45.68	11.13	28	3.9%	2.23 [1.52, 2.93]	2016
Dong,Q.2016	56.72	5.4	26	36.32	2.5	26	3.5%	4.78 [3.68, 5.87]	2016
Zhou,S.2017	59.46	7.64	43	55.53	7.69	43	4.2%	0.51 [0.08, 0.94]	2017
Wang,Y.2017	74.26	5.18	29	52.05	6.39	30	3.7%	3.76 [2.89, 4.63]	2017
**Subtotal (95% CI)**			**644**			**651**	**75.0%**	**2.30 [1.76, 2.85]**	
Heterogeneity: Tau² = 1.34; Chi² = 271.30, df = 18 (P < 0.00001); I² = 93%									
Test for overall effect: Z = 8.30 (P < 0.00001)									
**Ag-DCs-CIK cells**									
Shi,S.2012	60.1	12.1	30	49.9	12.7	30	4.1%	0.81 [0.28, 1.34]	2012
Zhu,Y.2013	58.02	4.97	30	59.68	7.37	31	4.1%	-0.26 [-0.76, 0.24]	2013
Zhang,M.2014	59.29	8.13	136	42.04	9.39	136	4.3%	1.96 [1.67, 2.25]	2014
Zhang, Z.2016	66.94	11.35	60	66.32	14.63	60	4.2%	0.05 [-0.31, 0.40]	2016
Yang, J.2016	70.54	12.84	60	51.64	10.61	60	4.2%	1.59 [1.18, 2.01]	2016
Zhao,H.2017	58.88	7.3	30	49.97	8.66	30	4.1%	1.10 [0.55, 1.64]	2017
**Subtotal (95% CI)**			**346**			**347**	**25.0%**	**0.88 [0.11, 1.65]**	
Heterogeneity: Tau² = 0.87; Chi² = 101.36, df = 5 (P < 0.00001); I² = 95%									
Test for overall effect: Z = 2.25 (P = 0.02)									
**Total (95% CI)**			**990**			**998**	**100.0%**	**1.95 [1.49, 2.40]**	
Heterogeneity: Tau² = 1.22; Chi² = 416.52, df = 24 (P < 0.00001); I² = 94%									
Test for overall effect: Z = 8.46 (P < 0.00001)									
Test for subgroup differences: Chi² = 8.79, df = 1 (P = 0.003), I² = 88.6%									

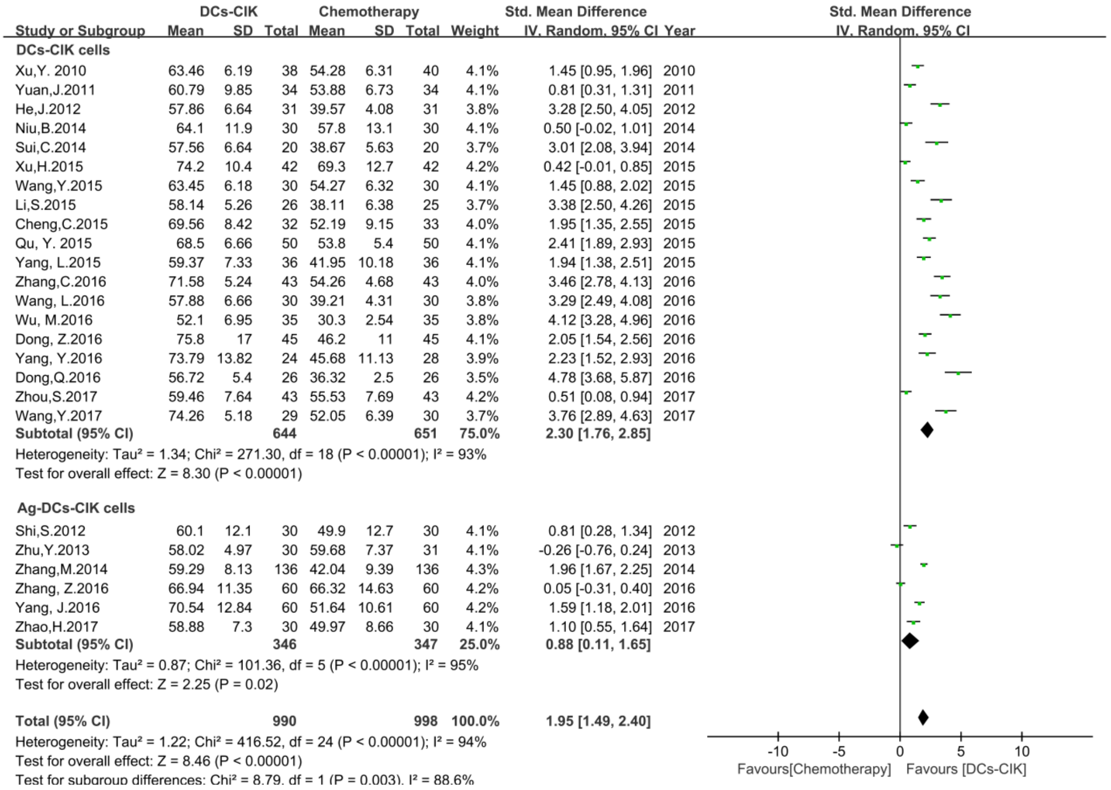

Figure S1.Subgroups analysis of $CD3^+$T cell via DCs-CIK types

Study or Subgroup	DCs-CIK Mean	DCs-CIK SD	DCs-CIK Total	Chemotherapy Mean	Chemotherapy SD	Chemotherapy Total	Weight	Std. Mean Difference IV, Random, 95% CI	Year
**DCs-CIK cells**									
Xu,Y. 2010	36.43	4.51	38	33.52	4.44	40	4.2%	0.64 [0.19, 1.10]	2010
Yuan,J.2011	34.81	7.07	34	29.38	4.99	34	4.0%	0.88 [0.38, 1.38]	2011
He,J.2012	30.29	9.12	31	23.15	3.82	31	3.9%	1.01 [0.48, 1.54]	2012
Zhao,L.2013	38.81	7.59	36	25.64	4.04	36	3.7%	2.14 [1.56, 2.73]	2013
Niu,B.2014	40.9	10.4	30	28.1	9.8	30	3.8%	1.25 [0.69, 1.81]	2014
Sui,C.2014	31.6	6.8	20	26.81	3.5	20	3.5%	0.87 [0.22, 1.52]	2014
Yang, L.2015	31.98	8.47	36	29.48	9.33	36	4.1%	0.28 [-0.19, 0.74]	2015
Wang,Y.2015	36.42	4.53	30	33.51	4.43	30	3.9%	0.64 [0.12, 1.16]	2015
Cheng,C.2015	35.78	3.33	40	26.18	4.87	40	3.8%	2.28 [1.71, 2.85]	2015
Xu,H.2015	32.2	11.5	42	24.5	12.4	42	4.2%	0.64 [0.20, 1.08]	2015
Qu, Y. 2015	22.7	3.6	50	22.8	2.7	50	4.4%	-0.03 [-0.42, 0.36]	2015
Li,S.2015	31.22	7.05	26	25.03	4.16	25	3.7%	1.05 [0.46, 1.64]	2015
Yang, Y.2016	42.05	8.99	24	23.76	11.22	28	3.5%	1.76 [1.11, 2.41]	2016
Wang, L.2016	30.3	9.12	30	23.15	3.64	30	3.9%	1.02 [0.48, 1.56]	2016
Wu, M.2016	20.8	6.82	35	16	3.53	35	4.0%	0.87 [0.38, 1.37]	2016
Dong, Z.2016	30.5	7	45	25.7	8	45	4.3%	0.63 [0.21, 1.06]	2016
Dong,Q.2016	36.17	5.59	26	30.16	2.84	26	3.6%	1.34 [0.73, 1.94]	2016
Wang,Y.2017	34.12	5.02	29	23.65	6.18	30	3.6%	1.83 [1.22, 2.45]	2017
Zhou,S.2017	36.12	9.18	43	35.27	8.14	43	4.3%	0.10 [-0.33, 0.52]	2017
**Subtotal (95% CI)**			**645**			**651**	**74.3%**	**0.99 [0.70, 1.27]**	
Heterogeneity: Tau² = 0.33; Chi² = 106.61, df = 18 (P < 0.00001); I² = 83%									
Test for overall effect: Z = 6.74 (P < 0.00001)									
**Ag-DCs-CIK cells**									
Shi,S.2012	40.1	9.6	30	32.3	9.4	30	3.9%	0.81 [0.28, 1.34]	2012
Zhu,Y.2013	36.26	5.31	30	33.32	6.58	31	4.0%	0.48 [-0.03, 0.99]	2013
Zhang,M.2014	32.59	7.37	136	28.79	7.15	136	4.9%	0.52 [0.28, 0.76]	2014
Yang, J.2016	39.51	12.82	60	32.25	13.46	60	4.5%	0.55 [0.18, 0.91]	2016
Zhang, Z.2016	34.86	9.33	60	28.8	11.32	60	4.5%	0.58 [0.21, 0.95]	2016
Zhao,H.2017	40.45	9.96	30	34.57	8.93	30	3.9%	0.61 [0.09, 1.13]	2017
**Subtotal (95% CI)**			**346**			**347**	**25.7%**	**0.57 [0.41, 0.72]**	
Heterogeneity: Tau² = 0.00; Chi² = 1.10, df = 5 (P = 0.95); I² = 0%									
Test for overall effect: Z = 7.29 (P < 0.00001)									
**Total (95% CI)**			**991**			**998**	**100.0%**	**0.88 [0.67, 1.09]**	
Heterogeneity: Tau² = 0.22; Chi² = 116.89, df = 24 (P < 0.00001); I² = 79%									
Test for overall effect: Z = 8.21 (P < 0.00001)									
Test for subgroup differences: Chi² = 6.46, df = 1 (P = 0.01), I² = 84.5%									

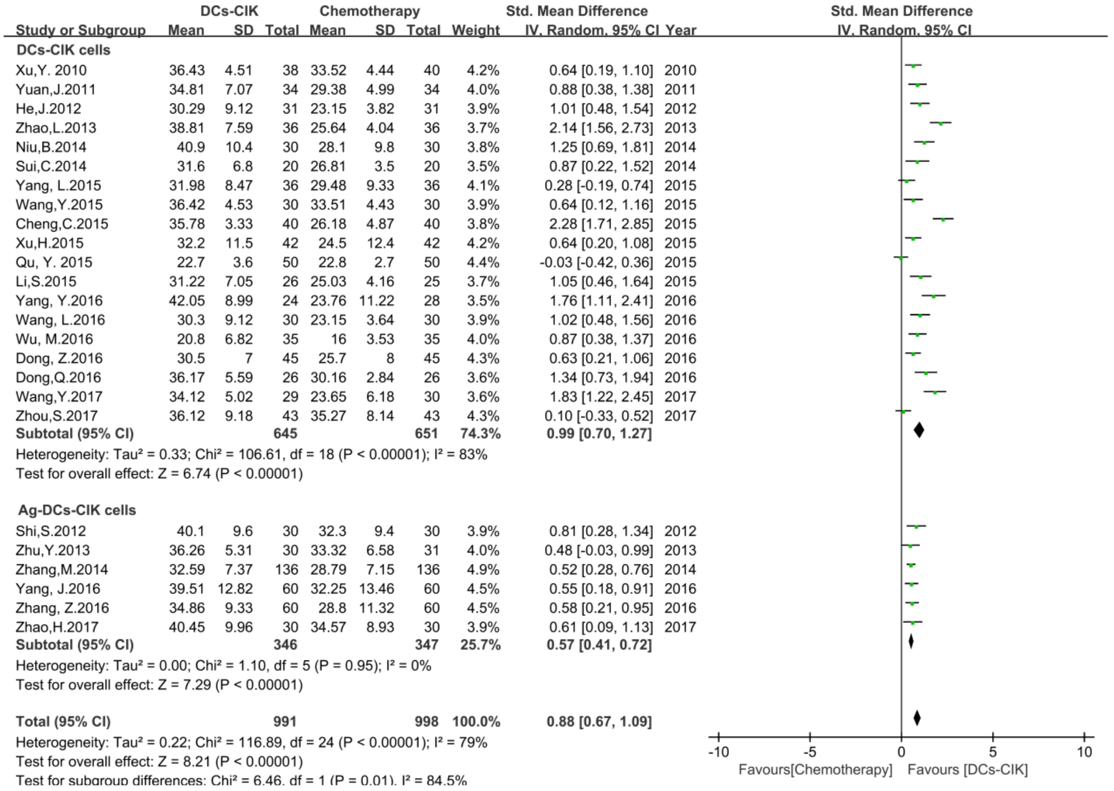

Figure S2.Subgroups analysis of $CD3^+$ $CD4^+$ T cell via DCs-CIK types

Study or Subgroup	DCs-CIK Mean	DCs-CIK SD	DCs-CIK Total	Chemotherapy Mean	Chemotherapy SD	Chemotherapy Total	Weight	Std. Mean Difference IV, Random, 95% CI	Year
**DCs-CIK cells**									
Xu,Y. 2010	26.12	4.21	38	26.72	5.36	40	3.7%	-0.12 [-0.57, 0.32]	2010
Yuan,J.2011	26.77	4.5	34	27.36	4.65	34	3.6%	-0.13 [-0.60, 0.35]	2011
Sheng,C.2011	38.13	5.76	32	22.69	5.07	33	3.5%	2.81 [2.12, 3.51]	2011
He,J.2012	25.68	4.46	31	23.87	5.16	31	3.6%	0.37 [-0.13, 0.87]	2012
Peng,D.2012	52.58	9.33	24	38.6	5.04	23	3.5%	1.82 [1.13, 2.51]	2012
Zhao,L.2013	24.19	5.73	36	25.28	4.19	36	3.6%	-0.21 [-0.68, 0.25]	2013
Han,W.2014	35.1	7.6	42	30.8	6.4	23	3.6%	0.59 [0.07, 1.11]	2014
Niu,B.2014	25.9	11.1	30	27.5	10.1	30	3.6%	-0.15 [-0.66, 0.36]	2014
Sui,C.2014	38.1	6.3	20	19.3	13.5	20	3.4%	1.75 [1.01, 2.49]	2014
Xu,H.2015	38.2	6.3	42	18.7	14.7	42	3.6%	1.71 [1.21, 2.21]	2015
Qu, Y. 2015	48.5	4.1	50	27.6	2.6	50	3.2%	6.04 [5.10, 6.98]	2015
Wang,Y.2015	26.11	4.22	30	26.72	5.35	30	3.6%	-0.12 [-0.63, 0.38]	2015
Yang, L.2015	27.14	9.36	36	20.17	9.33	36	3.6%	0.74 [0.26, 1.22]	2015
Cheng,C.2015	34.36	3.48	40	23.18	6.18	40	3.6%	2.21 [1.65, 2.77]	2015
Li,S.2015	24.42	5.32	26	23.61	5.26	25	3.6%	0.15 [-0.40, 0.70]	2015
Dong, Z.2016	37.4	9	45	19.7	5	45	3.6%	2.41 [1.86, 2.96]	2016
Wang, L.2016	25.68	4.45	30	23.65	5.64	30	3.6%	0.39 [-0.12, 0.91]	2016
Yang, Y.2016	29.58	6.48	24	19.89	10.48	28	3.6%	1.08 [0.49, 1.66]	2016
Wu, M.2016	31.3	4.56	35	14.4	1.8	35	3.2%	4.82 [3.87, 5.77]	2016
Dong,Q.2016	20.8	2.7	26	17.63	5.66	26	3.6%	0.70 [0.14, 1.27]	2016
Wang,Y.2017	37.52	6.21	29	19.21	6.53	30	3.4%	2.83 [2.10, 3.57]	2017
Zhou,S.2017	22.67	6.13	43	24.96	6.15	43	3.7%	-0.37 [-0.80, 0.06]	2017
**Subtotal (95% CI)**			**743**			**730**	**78.0%**	**1.29 [0.74, 1.84]**	
Heterogeneity: Tau² = 1.61; Chi² = 451.63, df = 21 (P < 0.00001); I² = 95%									
Test for overall effect: Z = 4.63 (P < 0.00001)									
**Ag-DCs-CIK cells**									
Shi,S.2012	27.4	5.1	30	33.1	12.4	30	3.6%	-0.59 [-1.11, -0.08]	2012
Zhu,Y.2013	28.21	5.63	30	26.33	5.82	31	3.6%	0.32 [-0.18, 0.83]	2013
Zhang,M.2014	26.7	3.75	136	20.25	5.8	136	3.7%	1.32 [1.05, 1.58]	2014
Yang, J.2016	28.78	8.36	60	19.83	9.77	60	3.7%	0.98 [0.60, 1.36]	2016
Zhang, Z.2016	30.7	10.09	60	34.67	13.48	60	3.7%	-0.33 [-0.69, 0.03]	2016
Zhao,H.2017	26.27	8.56	30	32.79	9.68	30	3.6%	-0.70 [-1.23, -0.18]	2017
**Subtotal (95% CI)**			**346**			**347**	**22.0%**	**0.18 [-0.56, 0.92]**	
Heterogeneity: Tau² = 0.80; Chi² = 101.73, df = 5 (P < 0.00001); I² = 95%									
Test for overall effect: Z = 0.48 (P = 0.63)									
**Total (95% CI)**			**1089**			**1077**	**100.0%**	**1.04 [0.60, 1.47]**	
Heterogeneity: Tau² = 1.30; Chi² = 565.17, df = 27 (P < 0.00001); I² = 95%									
Test for overall effect: Z = 4.67 (P < 0.00001)									
Test for subgroup differences: Chi² = 5.64, df = 1 (P = 0.02), I² = 82.3%									

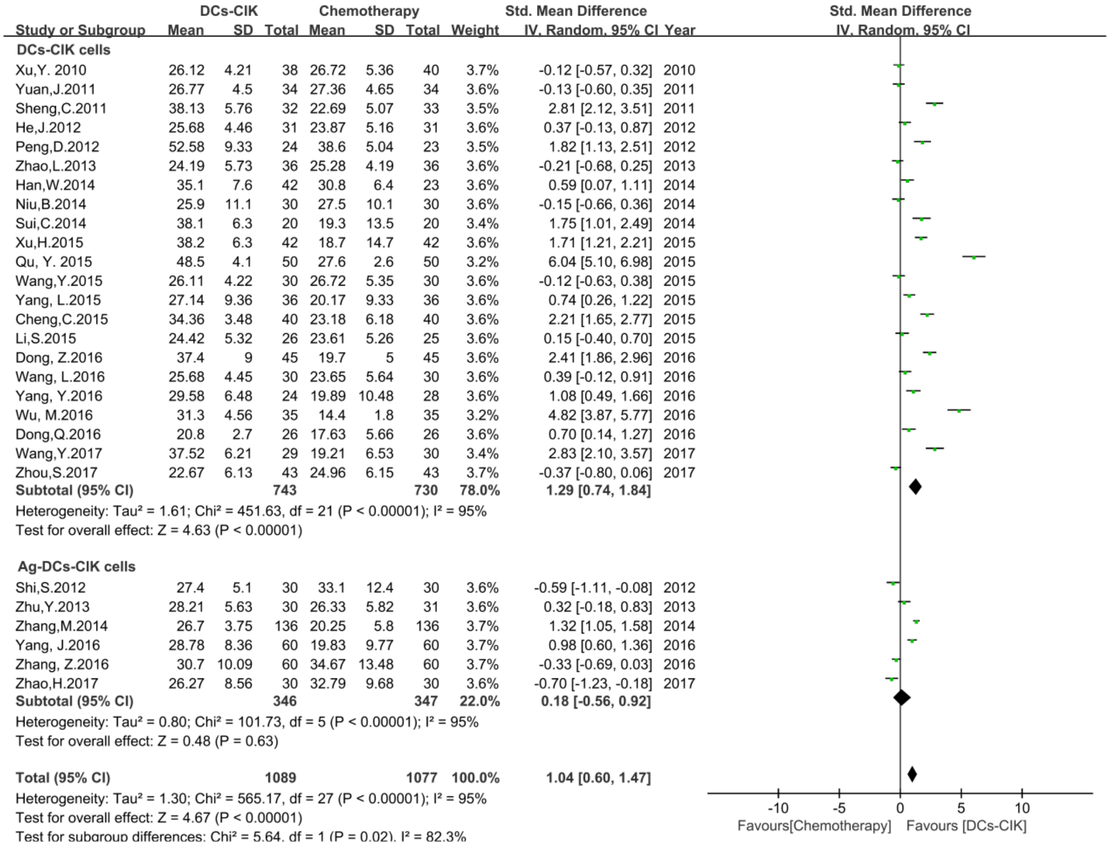

Figure S3.Subgroups analysis of $CD3^+$ $CD8^+$ T cell via DCs-CIK types

Study or Subgroup	DCs-CIK Mean	DCs-CIK SD	DCs-CIK Total	Chemotherapy Mean	Chemotherapy SD	Chemotherapy Total	Weight	Std. Mean Difference IV, Random, 95% CI	Year
**DCs-CIK cells**									
Xu,Y. 2010	1.64	0.35	38	1.11	0.37	40	6.7%	1.46 [0.95, 1.96]	2010
Yuan,J.2011	1.91	0.63	34	1.47	0.46	34	6.7%	0.79 [0.29, 1.28]	2011
He,J.2012	2.09	0.72	31	1.68	0.36	31	6.7%	0.71 [0.20, 1.23]	2012
Zhao,L.2013	1.7	0.58	36	1.04	0.23	36	6.6%	1.48 [0.96, 2.00]	2013
Niu,B.2014	1.3	0.2	30	1	0.1	30	6.4%	1.87 [1.26, 2.49]	2014
Qu, Y. 2015	0.49	0.16	50	0.83	0.38	50	6.9%	-1.16 [-1.58, -0.73]	2015
Li,S.2015	1.8	0.61	26	1.54	0.42	25	6.6%	0.49 [-0.07, 1.04]	2015
Wang,Y.2015	1.64	0.35	30	1.11	0.37	30	6.5%	1.45 [0.88, 2.03]	2015
Dong,Q.2016	1.75	0.26	26	1.91	0.28	26	6.6%	-0.58 [-1.14, -0.03]	2016
Yang, Y.2016	1.46	0.78	24	0.91	0.57	28	6.5%	0.80 [0.23, 1.37]	2016
Zhang,C.2016	2.03	0.77	43	0.86	0.32	43	6.7%	1.97 [1.45, 2.49]	2016
Wang, L.2016	2.08	0.73	30	1.66	0.35	30	6.6%	0.72 [0.20, 1.25]	2016
Zhou,S.2017	1.59	0.23	43	1.41	0.18	43	6.8%	0.86 [0.42, 1.31]	2017
**Subtotal (95% CI)**			**441**			**446**	**86.3%**	**0.83 [0.31, 1.35]**	
Heterogeneity: Tau² = 0.83; Chi² = 155.52, df = 12 (P < 0.00001); I² = 92%									
Test for overall effect: Z = 3.15 (P = 0.002)									
**Ag-DCs-CIK cells**									
Zhu,Y.2013	1.07	0.31	30	1.06	0.11	31	6.7%	0.04 [-0.46, 0.54]	2013
Zhang, Z.2016	1.26	0.6	60	1.02	0.75	60	7.0%	0.35 [-0.01, 0.71]	2016
**Subtotal (95% CI)**			**90**			**91**	**13.7%**	**0.25 [-0.05, 0.54]**	
Heterogeneity: Tau² = 0.00; Chi² = 0.96, df = 1 (P = 0.33); I² = 0%									
Test for overall effect: Z = 1.65 (P = 0.10)									
**Total (95% CI)**			**531**			**537**	**100.0%**	**0.74 [0.30, 1.19]**	
Heterogeneity: Tau² = 0.71; Chi² = 165.67, df = 14 (P < 0.00001); I² = 92%									
Test for overall effect: Z = 3.27 (P = 0.001)									
Test for subgroup differences: Chi² = 3.71, df = 1 (P = 0.05), I² = 73.0%									

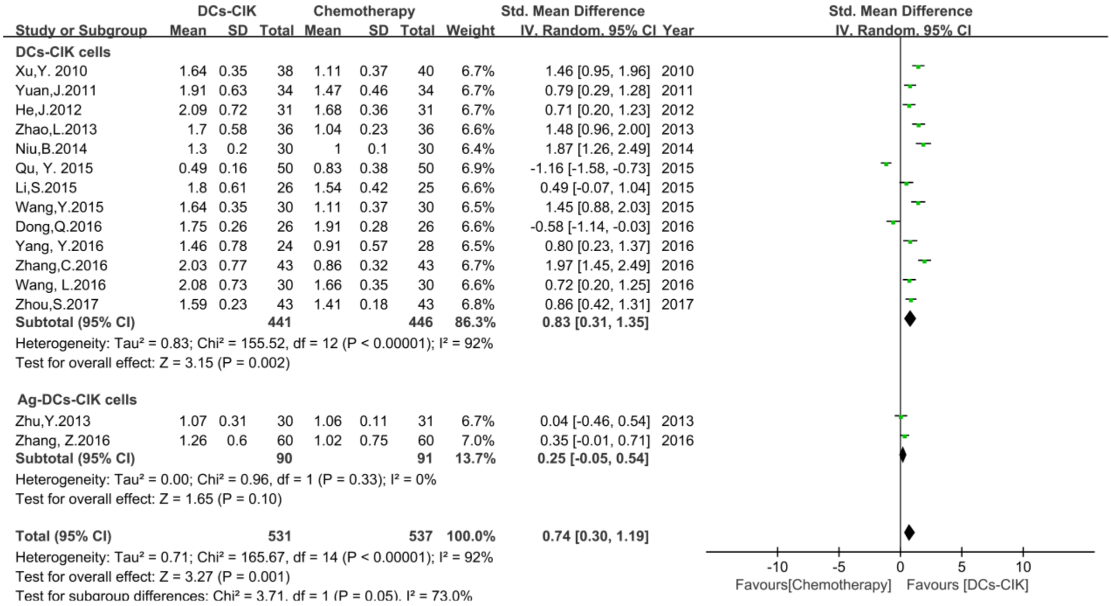

Figure S4.Subgroups analysis of $CD4^+/CD8^+$T cell via DCs-CIK types

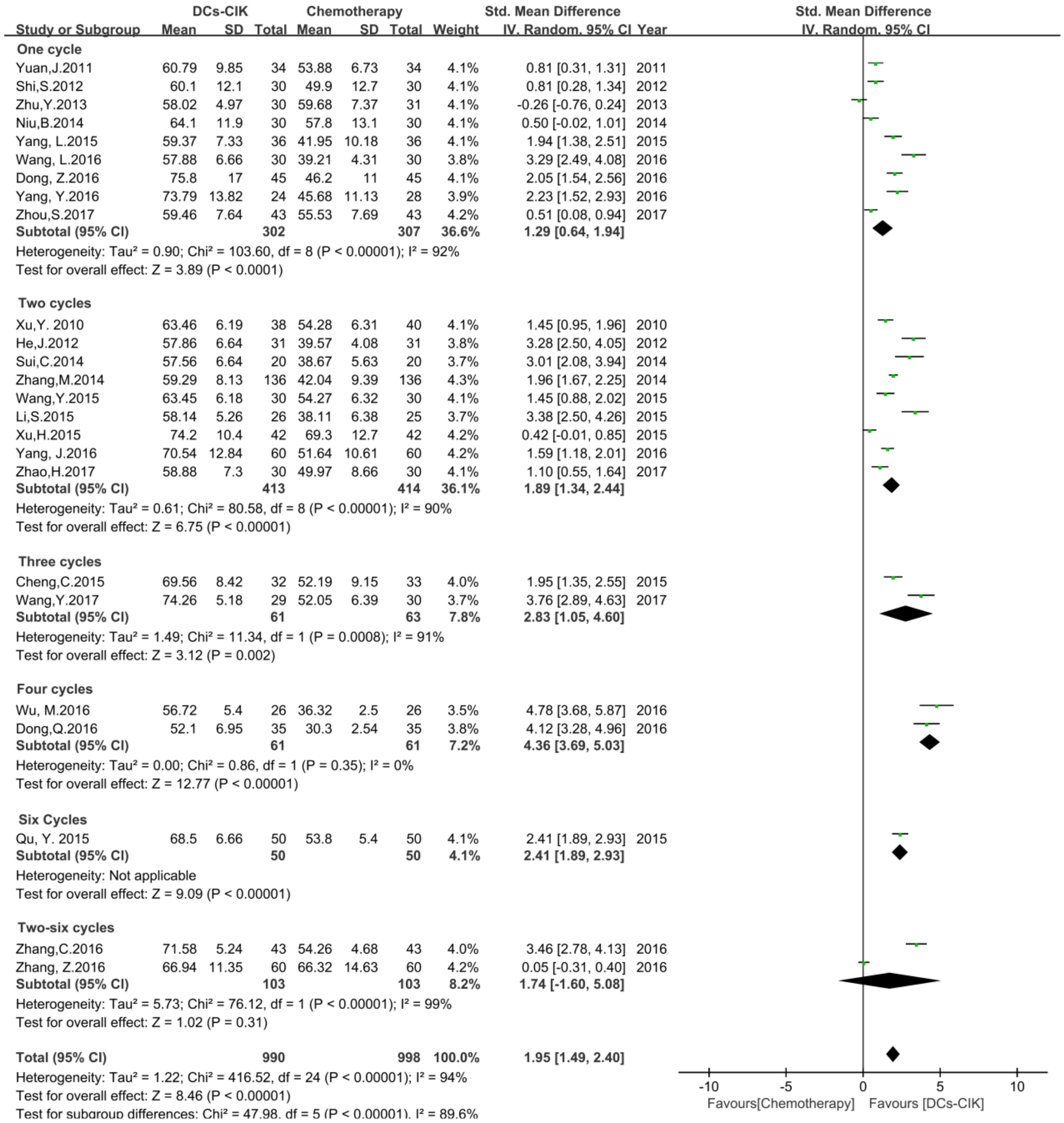

Figure S5.Subgroups analysis of $CD3^{+}$ T cell via treatment cycles

Study or Subgroup	DCs-CIK Mean	DCs-CIK SD	DCs-CIK Total	Chemotherapy Mean	Chemotherapy SD	Chemotherapy Total	Weight	Std. Mean Difference IV, Random, 95% CI	Year
**One cycle**									
Yuan,J.2011	34.81	7.07	34	29.38	4.99	34	4.0%	0.88 [0.38, 1.38]	2011
Shi,S.2012	40.1	9.6	30	32.3	9.4	30	3.9%	0.81 [0.28, 1.34]	2012
Zhu,Y.2013	36.26	5.31	30	33.32	6.58	31	4.0%	0.48 [-0.03, 0.99]	2013
Niu,B.2014	40.9	10.4	30	28.1	9.8	30	3.8%	1.25 [0.69, 1.81]	2014
Yang, L.2015	31.98	8.47	36	29.48	9.33	36	4.1%	0.28 [-0.19, 0.74]	2015
Yang, Y.2016	42.05	8.99	24	23.76	11.22	28	3.5%	1.76 [1.11, 2.41]	2016
Wang, L.2016	30.3	9.12	30	23.15	3.64	30	3.9%	1.02 [0.48, 1.56]	2016
Dong, Z.2016	30.5	7	45	25.7	8	45	4.3%	0.63 [0.21, 1.06]	2016
Zhou,S.2017	36.12	9.18	43	35.27	8.14	43	4.3%	0.10 [-0.33, 0.52]	2017
**Subtotal (95% CI)**			**302**			**307**	**35.7%**	**0.77 [0.46, 1.08]**	
Heterogeneity: Tau² = 0.16; Chi² = 27.69, df = 8 (P = 0.0005); I² = 71%									
Test for overall effect: Z = 4.82 (P < 0.00001)									
**Two cycles**									
Xu,Y. 2010	36.43	4.51	38	33.52	4.44	40	4.2%	0.64 [0.19, 1.10]	2010
He,J.2012	30.29	9.12	31	23.15	3.82	31	3.9%	1.01 [0.48, 1.54]	2012
Zhao,L.2013	38.81	7.59	36	25.64	4.04	36	3.7%	2.14 [1.56, 2.73]	2013
Zhang,M.2014	32.59	7.37	136	28.79	7.15	136	4.9%	0.52 [0.28, 0.76]	2014
Sui,C.2014	31.6	6.8	20	26.81	3.5	20	3.5%	0.87 [0.22, 1.52]	2014
Xu,H.2015	32.2	11.5	42	24.5	12.4	42	4.2%	0.64 [0.20, 1.08]	2015
Li,S.2015	31.22	7.05	26	25.03	4.16	25	3.7%	1.05 [0.46, 1.64]	2015
Wang,Y.2015	36.42	4.53	30	33.51	4.43	30	3.9%	0.64 [0.12, 1.16]	2015
Yang, J.2016	39.51	12.82	60	32.25	13.46	60	4.5%	0.55 [0.18, 0.91]	2016
Zhao,H.2017	40.45	9.96	30	34.57	8.93	30	3.9%	0.61 [0.09, 1.13]	2017
**Subtotal (95% CI)**			**449**			**450**	**40.4%**	**0.83 [0.57, 1.09]**	
Heterogeneity: Tau² = 0.12; Chi² = 29.11, df = 9 (P = 0.0006); I² = 69%									
Test for overall effect: Z = 6.22 (P < 0.00001)									
**Three cycles**									
Cheng,C.2015	35.78	3.33	40	26.18	4.87	40	3.8%	2.28 [1.71, 2.85]	2015
Wang,Y.2017	34.12	5.02	29	23.65	6.18	30	3.6%	1.83 [1.22, 2.45]	2017
**Subtotal (95% CI)**			**69**			**70**	**7.3%**	**2.07 [1.63, 2.51]**	
Heterogeneity: Tau² = 0.01; Chi² = 1.10, df = 1 (P = 0.30); I² = 9%									
Test for overall effect: Z = 9.29 (P < 0.00001)									
**Four cycles**									
Dong,Q.2016	36.17	5.59	26	30.16	2.84	26	3.6%	1.34 [0.73, 1.94]	2016
Wu, M.2016	20.8	6.82	35	16	3.53	35	4.0%	0.87 [0.38, 1.37]	2016
**Subtotal (95% CI)**			**61**			**61**	**7.7%**	**1.07 [0.62, 1.52]**	
Heterogeneity: Tau² = 0.03; Chi² = 1.34, df = 1 (P = 0.25); I² = 25%									
Test for overall effect: Z = 4.70 (P < 0.00001)									
**Two-six cycles**									
Zhang, Z.2016	34.86	9.33	60	28.8	11.32	60	4.5%	0.58 [0.21, 0.95]	2016
**Subtotal (95% CI)**			**60**			**60**	**4.5%**	**0.58 [0.21, 0.95]**	
Heterogeneity: Not applicable									
Test for overall effect: Z = 3.11 (P = 0.002)									
**Six Cycles**									
Qu, Y. 2015	22.7	3.6	50	22.8	2.7	50	4.4%	-0.03 [-0.42, 0.36]	2015
**Subtotal (95% CI)**			**50**			**50**	**4.4%**	**-0.03 [-0.42, 0.36]**	
Heterogeneity: Not applicable									
Test for overall effect: Z = 0.16 (P = 0.88)									
**Total (95% CI)**			**991**			**998**	**100.0%**	**0.88 [0.67, 1.09]**	
Heterogeneity: Tau² = 0.22; Chi² = 116.89, df = 24 (P < 0.00001); I² = 79%									
Test for overall effect: Z = 8.21 (P < 0.00001)									
Test for subgroup differences: Chi² = 52.54, df = 5 (P < 0.00001), I² = 90.5%									

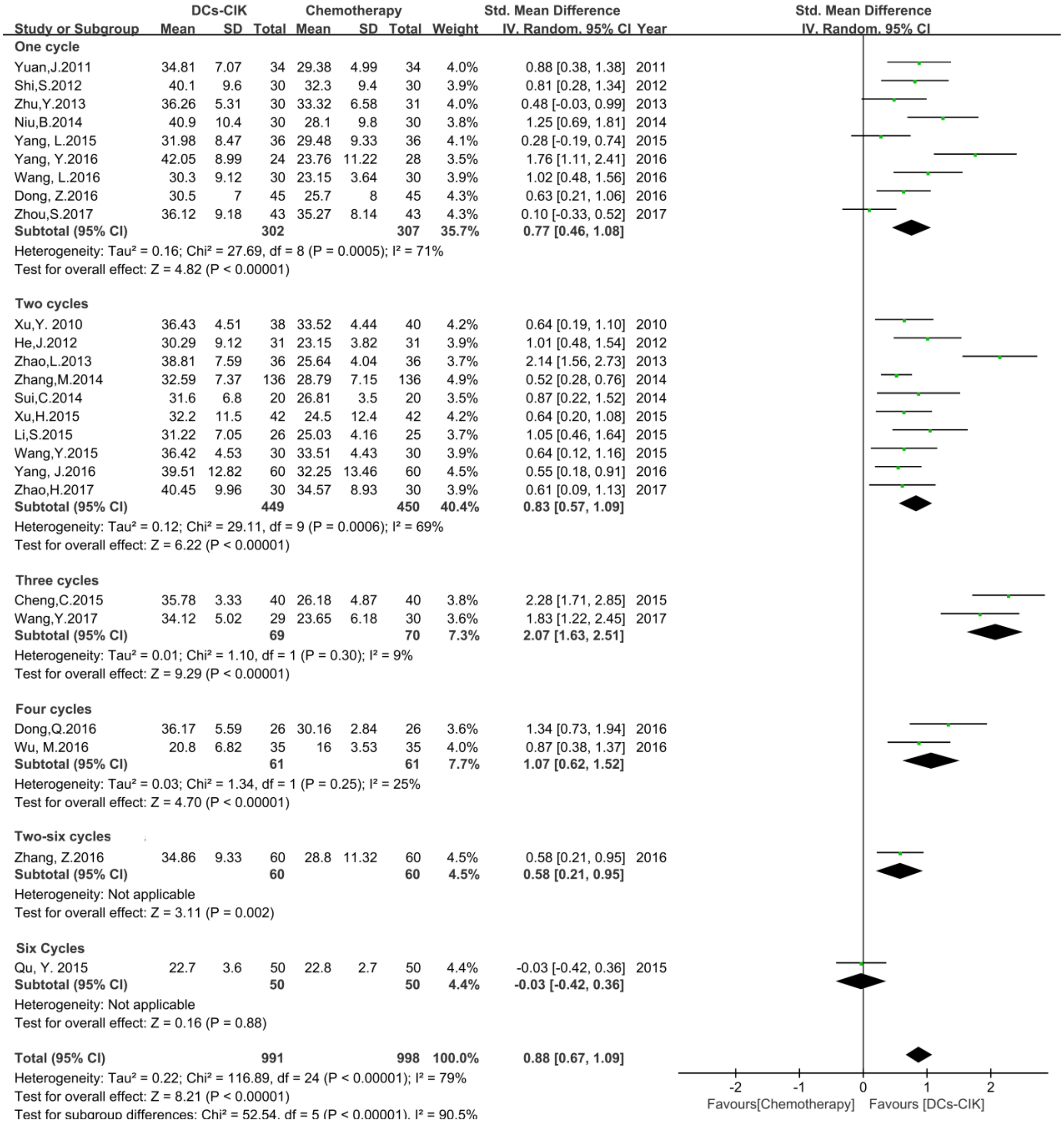

Figure S6.Subgroups analysis of $CD3^{+}$ $CD4^{+}$ T cell via treatment cycles

Study or Subgroup	DCs-CIK Mean	DCs-CIK SD	DCs-CIK Total	Chemotherapy Mean	Chemotherapy SD	Chemotherapy Total	Weight	Std. Mean Difference IV, Random, 95% CI	Year
**One cycle**									
Xu,Y. 2010	26.12	4.21	38	26.72	5.36	40	3.7%	-0.12 [-0.57, 0.32]	2010
Sheng,C.2011	38.13	5.76	32	22.69	5.07	33	3.5%	2.81 [2.12, 3.51]	2011
He,J.2012	25.68	4.46	31	23.87	5.16	31	3.6%	0.37 [-0.13, 0.87]	2012
Zhao,L.2013	24.19	5.73	36	25.28	4.19	36	3.6%	-0.21 [-0.68, 0.25]	2013
Sui,C.2014	38.1	6.3	20	19.3	13.5	20	3.4%	1.75 [1.01, 2.49]	2014
Zhang,M.2014	26.7	3.75	136	20.25	5.8	136	3.7%	1.32 [1.05, 1.58]	2014
Xu,H.2015	38.2	6.3	42	18.7	14.7	42	3.6%	1.71 [1.21, 2.21]	2015
Wang,Y.2015	26.11	4.22	30	26.72	5.35	30	3.6%	-0.12 [-0.63, 0.38]	2015
Li,S.2015	24.42	5.32	26	23.61	5.26	25	3.6%	0.15 [-0.40, 0.70]	2015
Yang, J.2016	28.78	8.36	60	19.83	9.77	60	3.7%	0.98 [0.60, 1.36]	2016
Zhao,H.2017	26.27	8.56	30	32.79	9.68	30	3.6%	-0.70 [-1.23, -0.18]	2017
**Subtotal (95% CI)**			**481**			**483**	**39.7%**	**0.70 [0.15, 1.25]**	
Heterogeneity: Tau² = 0.80; Chi² = 153.27, df = 10 (P < 0.00001); I² = 93%									
Test for overall effect: Z = 2.50 (P = 0.01)									
**Two cycles**									
Yuan,J.2011	26.77	4.5	34	27.36	4.65	34	3.6%	-0.13 [-0.60, 0.35]	2011
Peng,D.2012	52.58	9.33	24	38.6	5.04	23	3.5%	1.82 [1.13, 2.51]	2012
Shi,S.2012	27.4	5.1	30	33.1	12.4	30	3.6%	-0.59 [-1.11, -0.08]	2012
Zhu,Y.2013	28.21	5.63	30	26.33	5.82	31	3.6%	0.32 [-0.18, 0.83]	2013
Niu,B.2014	25.9	11.1	30	27.5	10.1	30	3.6%	-0.15 [-0.66, 0.36]	2014
Yang, L.2015	27.14	9.36	36	20.17	9.33	36	3.6%	0.74 [0.26, 1.22]	2015
Wang, L.2016	25.68	4.45	30	23.65	5.64	30	3.6%	0.39 [-0.12, 0.91]	2016
Dong, Z.2016	37.4	9	45	19.7	5	45	3.6%	2.41 [1.86, 2.96]	2016
Yang, Y.2016	29.58	6.48	24	19.89	10.48	28	3.6%	1.08 [0.49, 1.66]	2016
Zhou,S.2017	22.67	6.13	43	24.96	6.15	43	3.7%	-0.37 [-0.80, 0.06]	2017
**Subtotal (95% CI)**			**326**			**330**	**36.0%**	**0.54 [-0.03, 1.11]**	
Heterogeneity: Tau² = 0.78; Chi² = 111.01, df = 9 (P < 0.00001); I² = 92%									
Test for overall effect: Z = 1.84 (P = 0.07)									
**Three cycles**									
Cheng,C.2015	34.36	3.48	40	23.18	6.18	40	3.6%	2.21 [1.65, 2.77]	2015
Wang,Y.2017	37.52	6.21	29	19.21	6.53	30	3.4%	2.83 [2.10, 3.57]	2017
**Subtotal (95% CI)**			**69**			**70**	**7.0%**	**2.47 [1.87, 3.08]**	
Heterogeneity: Tau² = 0.08; Chi² = 1.76, df = 1 (P = 0.18); I² = 43%									
Test for overall effect: Z = 7.99 (P < 0.00001)									
**Four cycles**									
Han,W.2014	35.1	7.6	42	30.8	6.4	23	3.6%	0.59 [0.07, 1.11]	2014
Wu, M.2016	31.3	4.56	35	14.4	1.8	35	3.2%	4.82 [3.87, 5.77]	2016
Dong,Q.2016	20.8	2.7	26	17.63	5.66	26	3.6%	0.70 [0.14, 1.27]	2016
**Subtotal (95% CI)**			**103**			**84**	**10.4%**	**2.00 [-0.11, 4.11]**	
Heterogeneity: Tau² = 3.35; Chi² = 64.55, df = 2 (P < 0.00001); I² = 97%									
Test for overall effect: Z = 1.86 (P = 0.06)									
**Six Cycles**									
Qu, Y. 2015	48.5	4.1	50	27.6	2.6	50	3.2%	6.04 [5.10, 6.98]	2015
**Subtotal (95% CI)**			**50**			**50**	**3.2%**	**6.04 [5.10, 6.98]**	
Heterogeneity: Not applicable									
Test for overall effect: Z = 12.60 (P < 0.00001)									
**Two-six cycles**									
Zhang, Z.2016	30.7	10.09	60	34.67	13.48	60	3.7%	-0.33 [-0.69, 0.03]	2016
**Subtotal (95% CI)**			**60**			**60**	**3.7%**	**-0.33 [-0.69, 0.03]**	
Heterogeneity: Not applicable									
Test for overall effect: Z = 1.80 (P = 0.07)									
**Total (95% CI)**			**1089**			**1077**	**100.0%**	**1.04 [0.60, 1.47]**	
Heterogeneity: Tau² = 1.30; Chi² = 565.17, df = 27 (P < 0.00001); I² = 95%									
Test for overall effect: Z = 4.67 (P < 0.00001)									
Test for subgroup differences: Chi² = 188.51, df = 5 (P < 0.00001), I² = 97.3%									

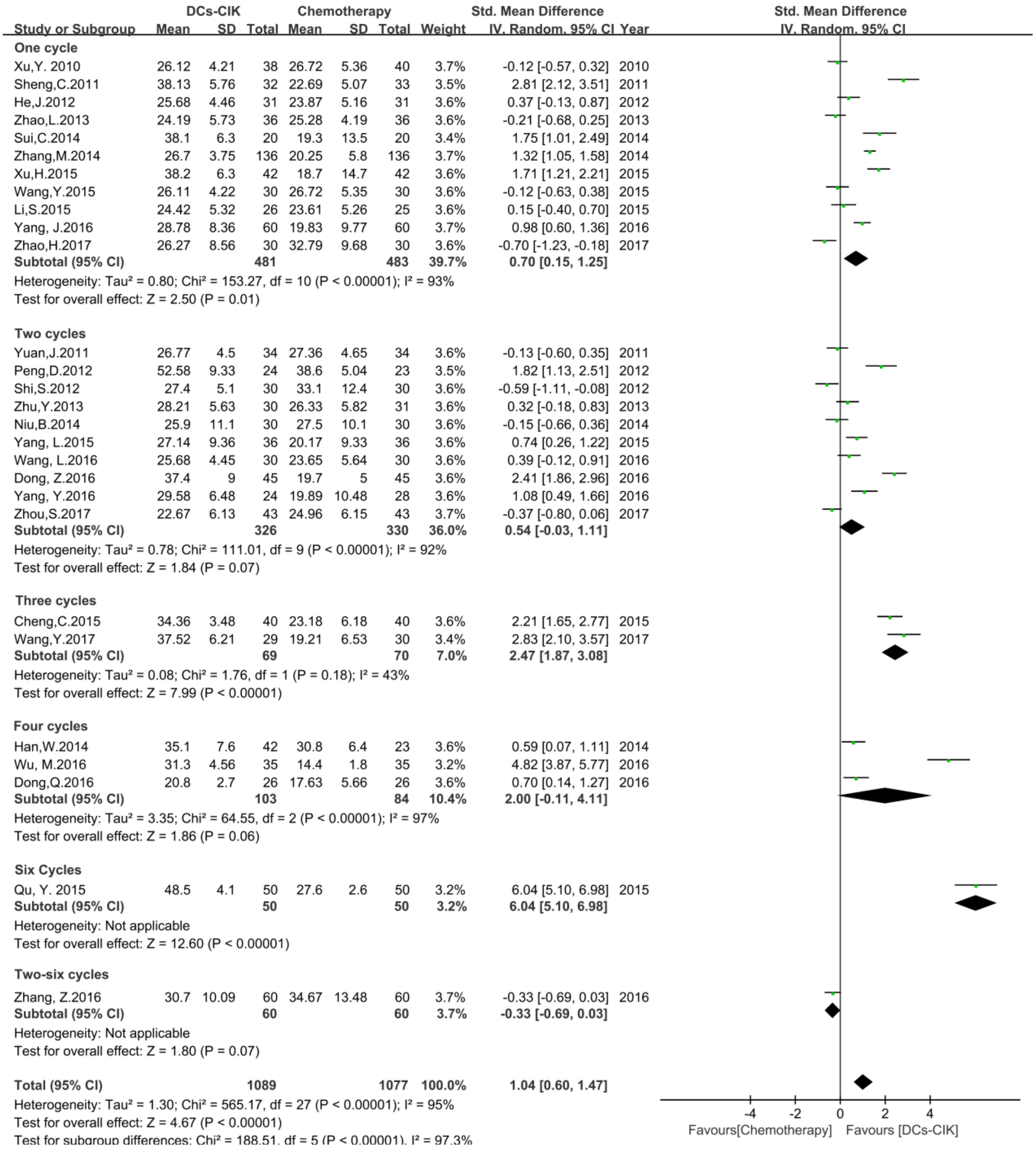

Figure S7.Subgroups analysis of $CD3^+$ $CD8^+$ T cell via treatment cycles

Study or Subgroup	DCs-CIK Mean	DCs-CIK SD	DCs-CIK Total	Chemotherapy Mean	Chemotherapy SD	Chemotherapy Total	Weight	Std. Mean Difference IV, Random, 95% CI	Year
**One cycle**									
Yuan,J.2011	1.91	0.63	34	1.47	0.46	34	6.7%	0.79 [0.29, 1.28]	2011
Zhu,Y.2013	1.07	0.31	30	1.06	0.11	31	6.7%	0.04 [-0.46, 0.54]	2013
Zhao,L.2013	1.7	0.58	36	1.04	0.23	36	6.6%	1.48 [0.96, 2.00]	2013
Niu,B.2014	1.3	0.2	30	1	0.1	30	6.4%	1.87 [1.26, 2.49]	2014
Yang, Y.2016	1.46	0.78	24	0.91	0.57	28	6.5%	0.80 [0.23, 1.37]	2016
Wang, L.2016	2.08	0.73	30	1.66	0.35	30	6.6%	0.72 [0.20, 1.25]	2016
Zhou,S.2017	1.59	0.23	43	1.41	0.18	43	6.8%	0.86 [0.42, 1.31]	2017
**Subtotal (95% CI)**			**227**			**232**	**46.5%**	**0.93 [0.51, 1.34]**	
Heterogeneity: Tau² = 0.24; Chi² = 26.31, df = 6 (P = 0.0002); I² = 77%									
Test for overall effect: Z = 4.41 (P < 0.0001)									
**Two cycles**									
Xu,Y. 2010	1.64	0.35	38	1.11	0.37	40	6.7%	1.46 [0.95, 1.96]	2010
He,J.2012	2.09	0.72	31	1.68	0.36	31	6.7%	0.71 [0.20, 1.23]	2012
Wang,Y.2015	1.64	0.35	30	1.11	0.37	30	6.5%	1.45 [0.88, 2.03]	2015
Li,S.2015	1.8	0.61	26	1.54	0.42	25	6.6%	0.49 [-0.07, 1.04]	2015
**Subtotal (95% CI)**			**125**			**126**	**26.5%**	**1.03 [0.54, 1.52]**	
Heterogeneity: Tau² = 0.17; Chi² = 9.97, df = 3 (P = 0.02); I² = 70%									
Test for overall effect: Z = 4.12 (P < 0.0001)									
**Four cycles**									
Dong,Q.2016	1.75	0.26	26	1.91	0.28	26	6.6%	-0.58 [-1.14, -0.03]	2016
**Subtotal (95% CI)**			**26**			**26**	**6.6%**	**-0.58 [-1.14, -0.03]**	
Heterogeneity: Not applicable									
Test for overall effect: Z = 2.06 (P = 0.04)									
**Six Cycles**									
Qu, Y. 2015	0.49	0.16	50	0.83	0.38	50	6.9%	-1.16 [-1.58, -0.73]	2015
**Subtotal (95% CI)**			**50**			**50**	**6.9%**	**-1.16 [-1.58, -0.73]**	
Heterogeneity: Not applicable									
Test for overall effect: Z = 5.34 (P < 0.00001)									
**Two-six cycles**									
Zhang, Z.2016	1.26	0.6	60	1.02	0.75	60	7.0%	0.35 [-0.01, 0.71]	2016
Zhang,C.2016	2.03	0.77	43	0.86	0.32	43	6.7%	1.97 [1.45, 2.49]	2016
**Subtotal (95% CI)**			**103**			**103**	**13.6%**	**1.15 [-0.44, 2.73]**	
Heterogeneity: Tau² = 1.25; Chi² = 25.11, df = 1 (P < 0.00001); I² = 96%									
Test for overall effect: Z = 1.42 (P = 0.16)									
**Total (95% CI)**			**531**			**537**	**100.0%**	**0.74 [0.30, 1.19]**	
Heterogeneity: Tau² = 0.71; Chi² = 165.67, df = 14 (P < 0.00001); I² = 92%									
Test for overall effect: Z = 3.27 (P = 0.001)									
Test for subgroup differences: Chi² = 70.46, df = 4 (P < 0.00001), I² = 94.3%									

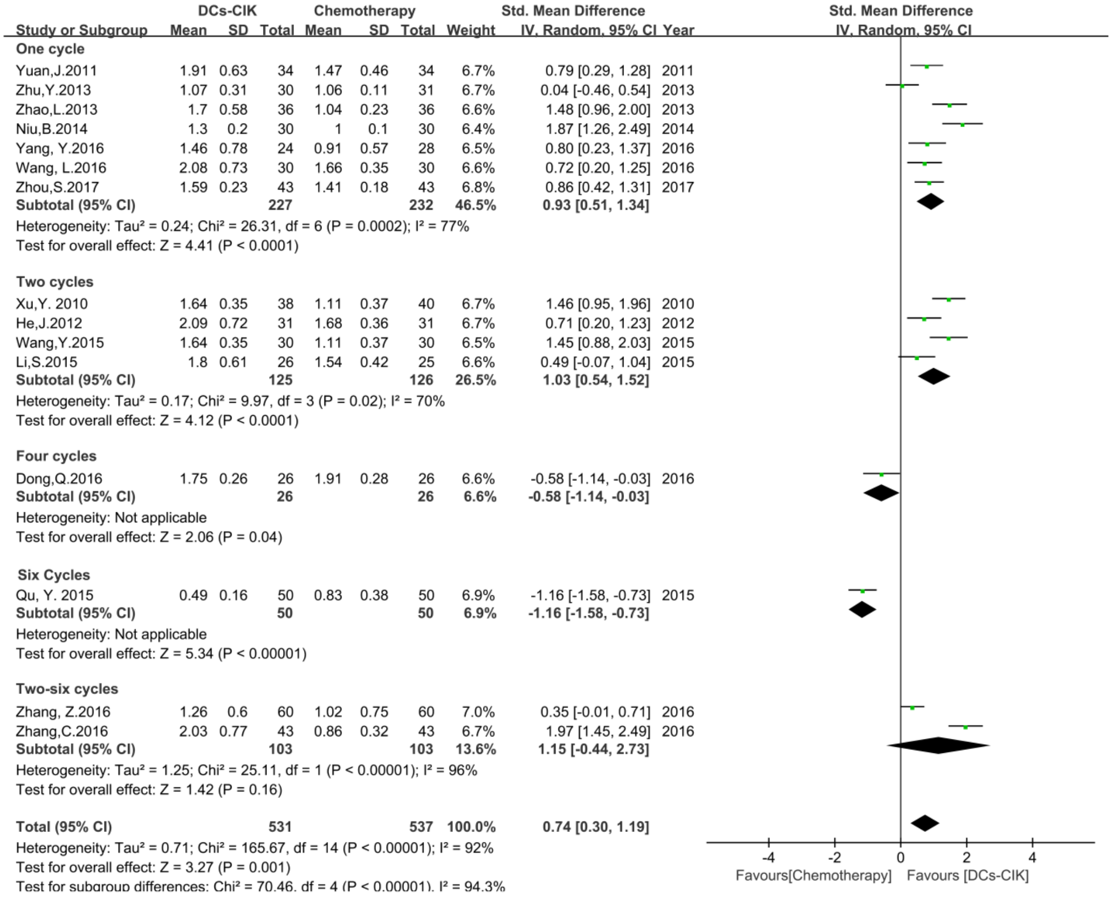

Figure S8.Subgroups analysis of $CD4^+/CD8^+$T cell via treatment cycles

Study or Subgroup	DCs-CIK Mean	SD	Total	Chemotherapy Mean	SD	Total	Weight	Std. Mean Difference IV, Random, 95% CI	Year
**Taxanes chemotherapy**									
Zhu,Y.2013	58.02	4.97	30	59.68	7.37	31	4.1%	-0.26 [-0.76, 0.24]	2013
Yang, L.2015	59.37	7.33	36	41.95	10.18	36	4.1%	1.94 [1.38, 2.51]	2015
Cheng,C.2015	69.56	8.42	32	52.19	9.15	33	4.0%	1.95 [1.35, 2.55]	2015
Qu, Y. 2015	68.5	6.66	50	53.8	5.4	50	4.1%	2.41 [1.89, 2.93]	2015
Xu,H.2015	74.2	10.4	42	69.3	12.7	42	4.2%	0.42 [-0.01, 0.85]	2015
Wu, M.2016	52.1	6.95	35	30.3	2.54	35	3.8%	4.12 [3.28, 4.96]	2016
Yang, Y.2016	73.79	13.82	24	45.68	11.13	28	3.9%	2.23 [1.52, 2.93]	2016
**Subtotal (95% CI)**			**249**			**255**	**28.2%**	**1.80 [0.81, 2.79]**	
Heterogeneity: Tau² = 1.69; Chi² = 127.72, df = 6 (P < 0.00001); I² = 95%									
Test for overall effect: Z = 3.57 (P = 0.0004)									
**Navebine and cisplatin**									
Xu,Y. 2010	63.46	6.19	38	54.28	6.31	40	4.1%	1.45 [0.95, 1.96]	2010
He,J.2012	57.86	6.64	31	39.57	4.08	31	3.8%	3.28 [2.50, 4.05]	2012
Sui,C.2014	57.56	6.64	20	38.67	5.63	20	3.7%	3.01 [2.08, 3.94]	2014
Wang,Y.2015	63.45	6.18	30	54.27	6.32	30	4.1%	1.45 [0.88, 2.02]	2015
Dong, Z.2016	75.8	17	45	46.2	11	45	4.1%	2.05 [1.54, 2.56]	2016
Wang, L.2016	57.88	6.66	30	39.21	4.31	30	3.8%	3.29 [2.49, 4.08]	2016
Wang,Y.2017	74.26	5.18	29	52.05	6.39	30	3.7%	3.76 [2.89, 4.63]	2017
**Subtotal (95% CI)**			**223**			**226**	**27.4%**	**2.56 [1.86, 3.26]**	
Heterogeneity: Tau² = 0.75; Chi² = 45.22, df = 6 (P < 0.00001); I² = 87%									
Test for overall effect: Z = 7.22 (P < 0.00001)									
**Gemcitabine and cisplatin**									
Niu,B.2014	64.1	11.9	30	57.8	13.1	30	4.1%	0.50 [-0.02, 1.01]	2014
Li,S.2015	58.14	5.26	26	38.11	6.38	25	3.7%	3.38 [2.50, 4.26]	2015
Zhang,C.2016	71.58	5.24	43	54.26	4.68	43	4.0%	3.46 [2.78, 4.13]	2016
Zhou,S.2017	59.46	7.64	43	55.53	7.69	43	4.2%	0.51 [0.08, 0.94]	2017
Zhao,H.2017	58.88	7.3	30	49.97	8.66	30	4.1%	1.10 [0.55, 1.64]	2017
**Subtotal (95% CI)**			**172**			**171**	**20.1%**	**1.75 [0.58, 2.93]**	
Heterogeneity: Tau² = 1.69; Chi² = 83.44, df = 4 (P < 0.00001); I² = 95%									
Test for overall effect: Z = 2.93 (P = 0.003)									
**Pemetrexed chemotherapy**									
Dong,Q.2016	56.72	5.4	26	36.32	2.5	26	3.5%	4.78 [3.68, 5.87]	2016
Yang, J.2016	70.54	12.84	60	51.64	10.61	60	4.2%	1.59 [1.18, 2.01]	2016
**Subtotal (95% CI)**			**86**			**86**	**7.6%**	**3.14 [0.03, 6.26]**	
Heterogeneity: Tau² = 4.88; Chi² = 28.22, df = 1 (P < 0.00001); I² = 96%									
Test for overall effect: Z = 1.98 (P = 0.05)									
**Systemic chemotherapy**									
Yuan,J.2011	60.79	9.85	34	53.88	6.73	34	4.1%	0.81 [0.31, 1.31]	2011
Shi,S.2012	60.1	12.1	30	49.9	12.7	30	4.1%	0.81 [0.28, 1.34]	2012
Zhang,M.2014	59.29	8.13	136	42.04	9.39	136	4.3%	1.96 [1.67, 2.25]	2014
Zhang, Z.2016	66.94	11.35	60	66.32	14.63	60	4.2%	0.05 [-0.31, 0.40]	2016
**Subtotal (95% CI)**			**260**			**260**	**16.7%**	**0.91 [-0.05, 1.87]**	
Heterogeneity: Tau² = 0.92; Chi² = 69.43, df = 3 (P < 0.00001); I² = 96%									
Test for overall effect: Z = 1.86 (P = 0.06)									
**Total (95% CI)**			**990**			**998**	**100.0%**	**1.95 [1.49, 2.40]**	
Heterogeneity: Tau² = 1.22; Chi² = 416.52, df = 24 (P < 0.00001); I² = 94%									
Test for overall effect: Z = 8.46 (P < 0.00001)									
Test for subgroup differences: Chi² = 8.19, df = 4 (P = 0.08), I² = 51.2%									

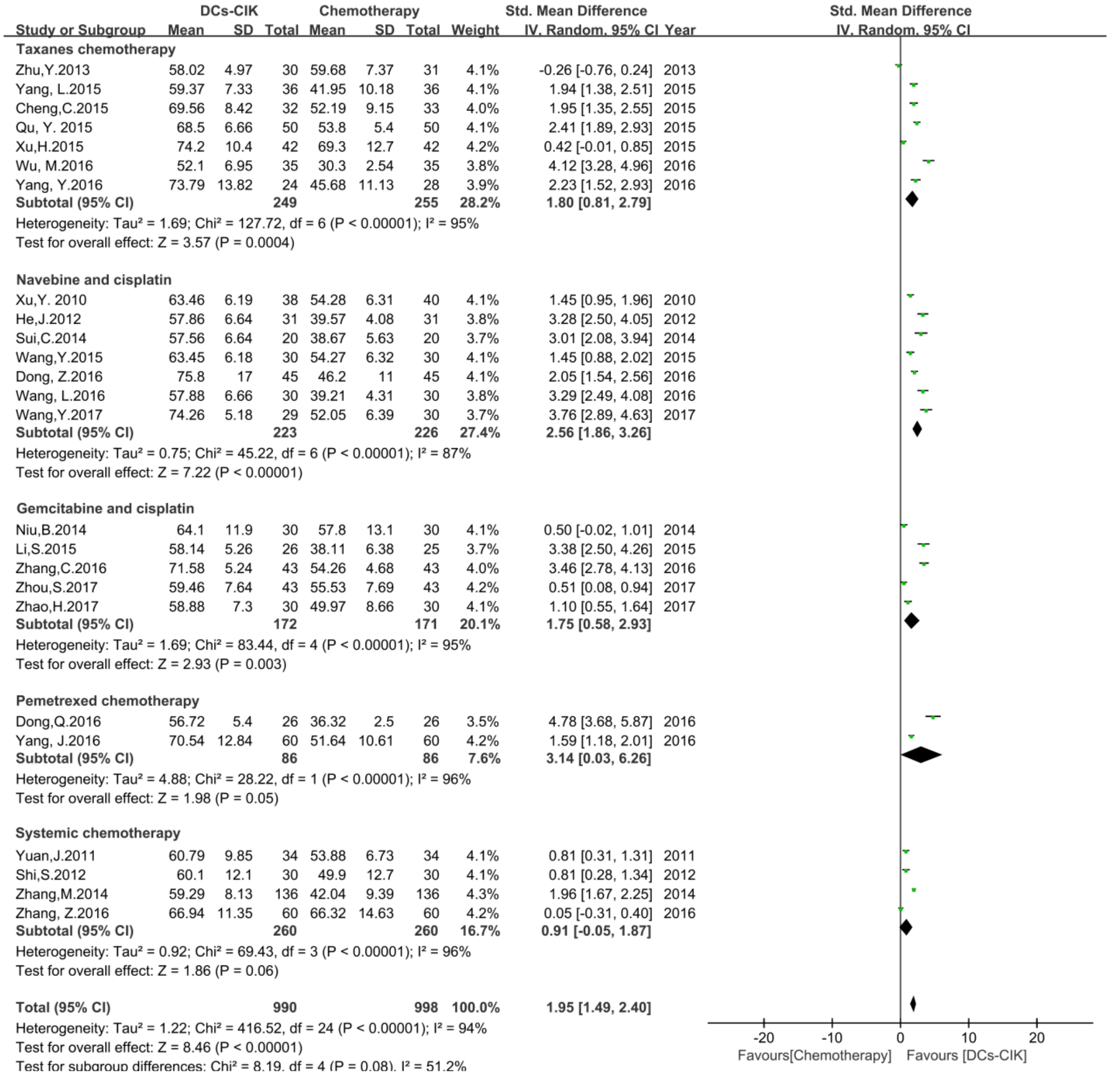

Figure S9.Subgroups analysis of $CD3^{+}$T cell via combinations with chemotherapy

Study or Subgroup	DCs-CIK Mean	DCs-CIK SD	DCs-CIK Total	Chemotherapy Mean	Chemotherapy SD	Chemotherapy Total	Weight	Std. Mean Difference IV, Random, 95% CI	Year
**Taxanes chemotherapy**									
Zhao,L.2013	38.81	7.59	36	25.64	4.04	36	3.7%	2.14 [1.56, 2.73]	2013
Zhu,Y.2013	36.26	5.31	30	33.32	6.58	31	4.0%	0.48 [-0.03, 0.99]	2013
Qu, Y. 2015	22.7	3.6	50	22.8	2.7	50	4.4%	-0.03 [-0.42, 0.36]	2015
Cheng,C.2015	35.78	3.33	40	26.18	4.87	40	3.8%	2.28 [1.71, 2.85]	2015
Xu,H.2015	32.2	11.5	42	24.5	12.4	42	4.2%	0.64 [0.20, 1.08]	2015
Yang, L.2015	31.98	8.47	36	29.48	9.33	36	4.1%	0.28 [-0.19, 0.74]	2015
Yang, Y.2016	42.05	8.99	24	23.76	11.22	28	3.5%	1.76 [1.11, 2.41]	2016
Wu, M.2016	20.8	6.82	35	16	3.53	35	4.0%	0.87 [0.38, 1.37]	2016
**Subtotal (95% CI)**			**293**			**298**	**31.7%**	**1.03 [0.44, 1.62]**	
Heterogeneity: Tau² = 0.66; Chi² = 78.60, df = 7 (P < 0.00001); I² = 91%									
Test for overall effect: Z = 3.42 (P = 0.0006)									
**Navebine and cisplatin**									
Xu,Y. 2010	36.43	4.51	38	33.52	4.44	40	4.2%	0.64 [0.19, 1.10]	2010
He,J.2012	30.29	9.12	31	23.15	3.82	31	3.9%	1.01 [0.48, 1.54]	2012
Sui,C.2014	31.6	6.8	20	26.81	3.5	20	3.5%	0.87 [0.22, 1.52]	2014
Wang,Y.2015	36.42	4.53	30	33.51	4.43	30	3.9%	0.64 [0.12, 1.16]	2015
Wang, L.2016	30.3	9.12	30	23.15	3.64	30	3.9%	1.02 [0.48, 1.56]	2016
Dong, Z.2016	30.5	7	45	25.7	8	45	4.3%	0.63 [0.21, 1.06]	2016
Wang,Y.2017	34.12	5.02	29	23.65	6.18	30	3.6%	1.83 [1.22, 2.45]	2017
**Subtotal (95% CI)**			**223**			**226**	**27.2%**	**0.92 [0.63, 1.21]**	
Heterogeneity: Tau² = 0.08; Chi² = 12.83, df = 6 (P = 0.05); I² = 53%									
Test for overall effect: Z = 6.19 (P < 0.00001)									
**Pemetrexed chemotherapy**									
Dong,Q.2016	36.17	5.59	26	30.16	2.84	26	3.6%	1.34 [0.73, 1.94]	2016
Yang, J.2016	39.51	12.82	60	32.25	13.46	60	4.5%	0.55 [0.18, 0.91]	2016
**Subtotal (95% CI)**			**86**			**86**	**8.1%**	**0.90 [0.14, 1.67]**	
Heterogeneity: Tau² = 0.24; Chi² = 4.75, df = 1 (P = 0.03); I² = 79%									
Test for overall effect: Z = 2.31 (P = 0.02)									
**Gemcitabine and cisplatin**									
Niu,B.2014	40.9	10.4	30	28.1	9.8	30	3.8%	1.25 [0.69, 1.81]	2014
Li,S.2015	31.22	7.05	26	25.03	4.16	25	3.7%	1.05 [0.46, 1.64]	2015
Zhou,S.2017	36.12	9.18	43	35.27	8.14	43	4.3%	0.10 [-0.33, 0.52]	2017
Zhao,H.2017	40.45	9.96	30	34.57	8.93	30	3.9%	0.61 [0.09, 1.13]	2017
**Subtotal (95% CI)**			**129**			**128**	**15.7%**	**0.73 [0.19, 1.26]**	
Heterogeneity: Tau² = 0.23; Chi² = 12.81, df = 3 (P = 0.005); I² = 77%									
Test for overall effect: Z = 2.68 (P = 0.007)									
**Systemic chemotherapy**									
Yuan,J.2011	34.81	7.07	34	29.38	4.99	34	4.0%	0.88 [0.38, 1.38]	2011
Shi,S.2012	40.1	9.6	30	32.3	9.4	30	3.9%	0.81 [0.28, 1.34]	2012
Zhang,M.2014	32.59	7.37	136	28.79	7.15	136	4.9%	0.52 [0.28, 0.76]	2014
Zhang, Z.2016	34.86	9.33	60	28.8	11.32	60	4.5%	0.58 [0.21, 0.95]	2016
**Subtotal (95% CI)**			**260**			**260**	**17.3%**	**0.61 [0.44, 0.79]**	
Heterogeneity: Tau² = 0.00; Chi² = 2.19, df = 3 (P = 0.53); I² = 0%									
Test for overall effect: Z = 6.81 (P < 0.00001)									
**Total (95% CI)**			**991**			**998**	**100.0%**	**0.88 [0.67, 1.09]**	
Heterogeneity: Tau² = 0.22; Chi² = 116.89, df = 24 (P < 0.00001); I² = 79%									
Test for overall effect: Z = 8.21 (P < 0.00001)									
Test for subgroup differences: Chi² = 4.50, df = 4 (P = 0.34), I² = 11.2%									

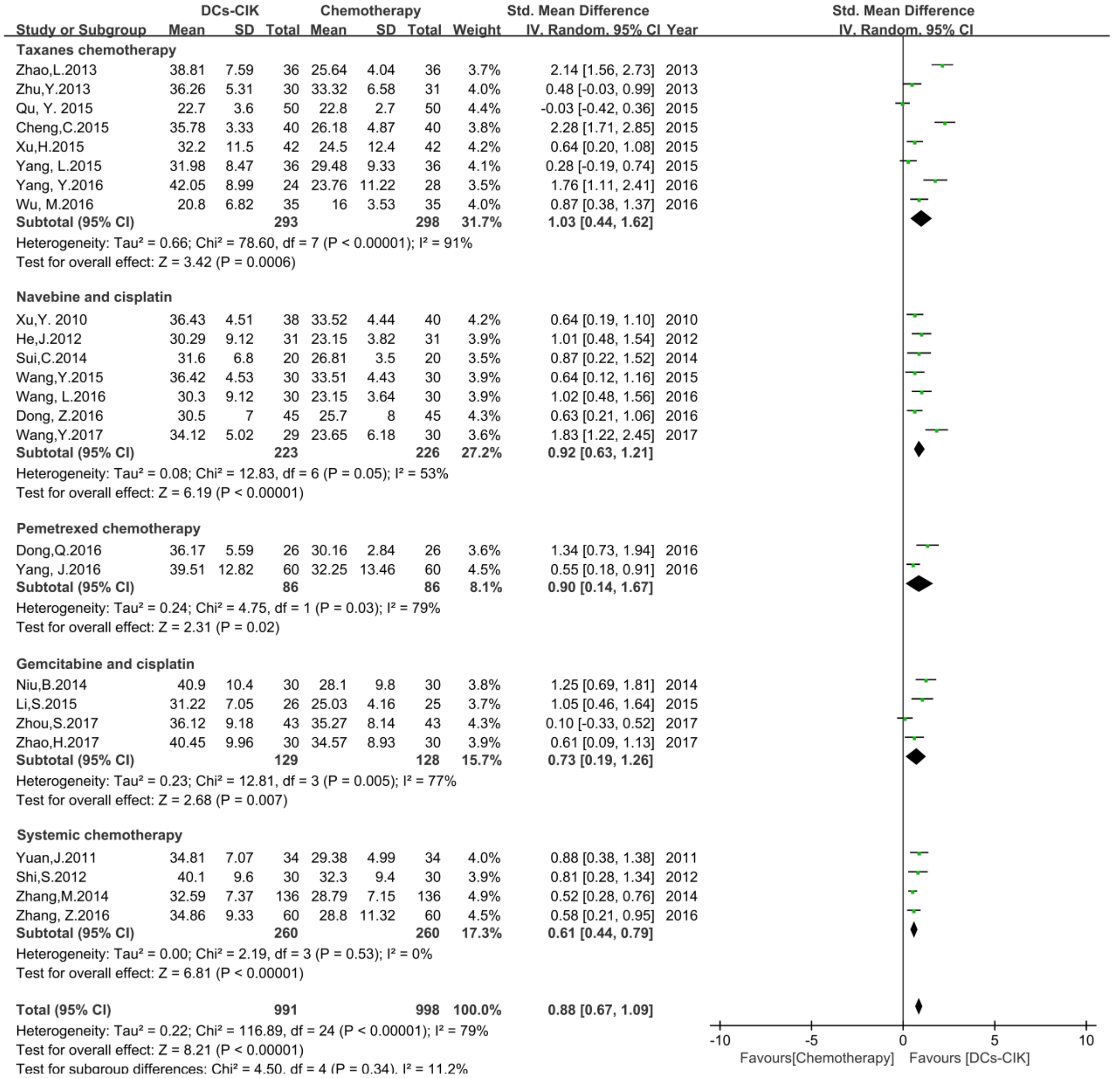

Figure S10.Subgroups analysis of $CD3^+$ $CD4^+$ T cell via combinations with chemotherapy

Study or Subgroup	DCs-CIK Mean	DCs-CIK SD	DCs-CIK Total	Chemotherapy Mean	Chemotherapy SD	Chemotherapy Total	Weight	Std. Mean Difference IV, Random, 95% CI	Year
**Taxanes chemotherapy**									
Zhu,Y.2013	28.21	5.63	30	26.33	5.82	31	3.6%	0.32 [-0.18, 0.83]	2013
Zhao,L.2013	24.19	5.73	36	25.28	4.19	36	3.6%	-0.21 [-0.68, 0.25]	2013
Han,W.2014	35.1	7.6	42	30.8	6.4	23	3.6%	0.59 [0.07, 1.11]	2014
Qu, Y. 2015	48.5	4.1	50	27.6	2.6	50	3.2%	6.04 [5.10, 6.98]	2015
Yang, L.2015	27.14	9.36	36	20.17	9.33	36	3.6%	0.74 [0.26, 1.22]	2015
Xu,H.2015	38.2	6.3	42	18.7	14.7	42	3.6%	1.71 [1.21, 2.21]	2015
Cheng,C.2015	34.36	3.48	40	23.18	6.18	40	3.6%	2.21 [1.65, 2.77]	2015
Wu, M.2016	31.3	4.56	35	14.4	1.8	35	3.2%	4.82 [3.87, 5.77]	2016
Yang, Y.2016	29.58	6.48	24	19.89	10.48	28	3.6%	1.08 [0.49, 1.66]	2016
**Subtotal (95% CI)**			**335**			**321**	**31.7%**	**1.87 [0.86, 2.88]**	
Heterogeneity: Tau² = 2.30; Chi² = 230.90, df = 8 (P < 0.00001); I² = 97%									
Test for overall effect: Z = 3.62 (P = 0.0003)									
**Navebine and cisplatin**									
Xu,Y. 2010	26.12	4.21	38	26.72	5.36	40	3.7%	-0.12 [-0.57, 0.32]	2010
Sheng,C.2011	38.13	5.76	32	22.69	5.07	33	3.5%	2.81 [2.12, 3.51]	2011
He,J.2012	25.68	4.46	31	23.87	5.16	31	3.6%	0.37 [-0.13, 0.87]	2012
Sui,C.2014	38.1	6.3	20	19.3	13.5	20	3.4%	1.75 [1.01, 2.49]	2014
Wang,Y.2015	26.11	4.22	30	26.72	5.35	30	3.6%	-0.12 [-0.63, 0.38]	2015
Dong, Z.2016	37.4	9	45	19.7	5	45	3.6%	2.41 [1.86, 2.96]	2016
Wang, L.2016	25.68	4.45	30	23.65	5.64	30	3.6%	0.39 [-0.12, 0.91]	2016
Wang,Y.2017	37.52	6.21	29	19.21	6.53	30	3.4%	2.83 [2.10, 3.57]	2017
**Subtotal (95% CI)**			**255**			**259**	**28.4%**	**1.27 [0.40, 2.14]**	
Heterogeneity: Tau² = 1.49; Chi² = 133.79, df = 7 (P < 0.00001); I² = 95%									
Test for overall effect: Z = 2.86 (P = 0.004)									
**Gemcitabine and cisplatin**									
Niu,B.2014	25.9	11.1	30	27.5	10.1	30	3.6%	-0.15 [-0.66, 0.36]	2014
Li,S.2015	24.42	5.32	26	23.61	5.26	25	3.6%	0.15 [-0.40, 0.70]	2015
Zhou,S.2017	22.67	6.13	43	24.96	6.15	43	3.7%	-0.37 [-0.80, 0.06]	2017
Zhao,H.2017	26.27	8.56	30	32.79	9.68	30	3.6%	-0.70 [-1.23, -0.18]	2017
**Subtotal (95% CI)**			**129**			**128**	**14.5%**	**-0.28 [-0.61, 0.05]**	
Heterogeneity: Tau² = 0.05; Chi² = 5.31, df = 3 (P = 0.15); I² = 44%									
Test for overall effect: Z = 1.65 (P = 0.10)									
**Pemetrexed chemotherapy**									
Peng,D.2012	52.58	9.33	24	38.6	5.04	23	3.5%	1.82 [1.13, 2.51]	2012
Dong,Q.2016	20.8	2.7	26	17.63	5.66	26	3.6%	0.70 [0.14, 1.27]	2016
Yang, J.2016	28.78	8.36	60	19.83	9.77	60	3.7%	0.98 [0.60, 1.36]	2016
**Subtotal (95% CI)**			**110**			**109**	**10.7%**	**1.12 [0.57, 1.68]**	
Heterogeneity: Tau² = 0.16; Chi² = 6.41, df = 2 (P = 0.04); I² = 69%									
Test for overall effect: Z = 3.99 (P < 0.0001)									
**Systemic chemotherapy**									
Yuan,J.2011	26.77	4.5	34	27.36	4.65	34	3.6%	-0.13 [-0.60, 0.35]	2011
Shi,S.2012	27.4	5.1	30	33.1	12.4	30	3.6%	-0.59 [-1.11, -0.08]	2012
Zhang,M.2014	26.7	3.75	136	20.25	5.8	136	3.7%	1.32 [1.05, 1.58]	2014
Zhang, Z.2016	30.7	10.09	60	34.67	13.48	60	3.7%	-0.33 [-0.69, 0.03]	2016
**Subtotal (95% CI)**			**260**			**260**	**14.7%**	**0.08 [-0.92, 1.08]**	
Heterogeneity: Tau² = 1.00; Chi² = 81.21, df = 3 (P < 0.00001); I² = 96%									
Test for overall effect: Z = 0.15 (P = 0.88)									
**Total (95% CI)**			**1089**			**1077**	**100.0%**	**1.04 [0.60, 1.47]**	
Heterogeneity: Tau² = 1.30; Chi² = 565.17, df = 27 (P < 0.00001); I² = 95%									
Test for overall effect: Z = 4.67 (P < 0.00001)									
Test for subgroup differences: Chi² = 34.42, df = 4 (P < 0.00001), I² = 88.4%									

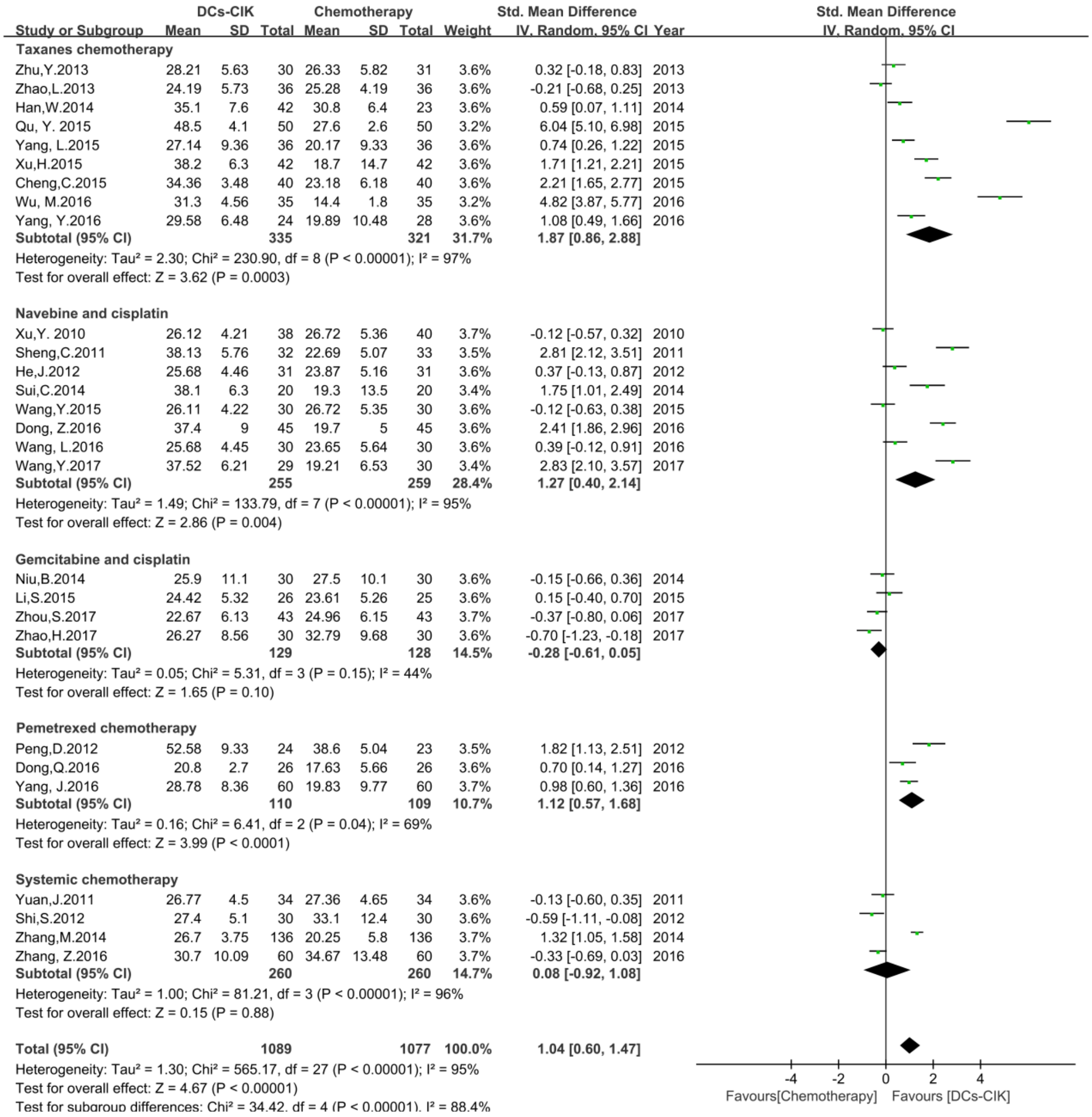

Figure S11.Subgroups analysis of $CD3^+$ $CD8^+$ T cell via combinations with chemotherapy

Study or Subgroup	DCs-CIK Mean	DCs-CIK SD	DCs-CIK Total	Chemotherapy Mean	Chemotherapy SD	Chemotherapy Total	Weight	Std. Mean Difference IV, Random, 95% CI
**Taxanes chemotherapy**								
Qu, Y. 2015	0.49	0.16	50	0.83	0.38	50	6.9%	-1.16 [-1.58, -0.73]
Zhu,Y.2013	1.07	0.31	30	1.06	0.11	31	6.7%	0.04 [-0.46, 0.54]
Yang, Y.2016	1.46	0.78	24	0.91	0.57	28	6.5%	0.80 [0.23, 1.37]
Zhao,L.2013	1.7	0.58	36	1.04	0.23	36	6.6%	1.48 [0.96, 2.00]
**Subtotal (95% CI)**			**140**			**145**	**26.7%**	**0.28 [-0.89, 1.46]**
Heterogeneity: Tau² = 1.37; Chi² = 66.15, df = 3 (P < 0.00001); I² = 95%								
Test for overall effect: Z = 0.47 (P = 0.64)								
**Navebine and cisplatin**								
He,J.2012	2.09	0.72	31	1.68	0.36	31	6.7%	0.71 [0.20, 1.23]
Wang, L.2016	2.08	0.73	30	1.66	0.35	30	6.6%	0.72 [0.20, 1.25]
Wang,Y.2015	1.64	0.35	30	1.11	0.37	30	6.5%	1.45 [0.88, 2.03]
Xu,Y. 2010	1.64	0.35	38	1.11	0.37	40	6.7%	1.46 [0.95, 1.96]
**Subtotal (95% CI)**			**129**			**131**	**26.5%**	**1.08 [0.66, 1.50]**
Heterogeneity: Tau² = 0.11; Chi² = 7.53, df = 3 (P = 0.06); I² = 60%								
Test for overall effect: Z = 5.07 (P < 0.00001)								
**Gemcitabine and cisplatin**								
Li,S.2015	1.8	0.61	26	1.54	0.42	25	6.6%	0.49 [-0.07, 1.04]
Zhou,S.2017	1.59	0.23	43	1.41	0.18	43	6.8%	0.86 [0.42, 1.31]
Niu,B.2014	1.3	0.2	30	1	0.1	30	6.4%	1.87 [1.26, 2.49]
Zhang,C.2016	2.03	0.77	43	0.86	0.32	43	6.7%	1.97 [1.45, 2.49]
**Subtotal (95% CI)**			**142**			**141**	**26.5%**	**1.29 [0.58, 2.00]**
Heterogeneity: Tau² = 0.44; Chi² = 21.39, df = 3 (P < 0.0001); I² = 86%								
Test for overall effect: Z = 3.58 (P = 0.0003)								
**Pemetrexed chemotherapy**								
Dong,Q.2016	1.75	0.26	26	1.91	0.28	26	6.6%	-0.58 [-1.14, -0.03]
**Subtotal (95% CI)**			**26**			**26**	**6.6%**	**-0.58 [-1.14, -0.03]**
Heterogeneity: Not applicable								
Test for overall effect: Z = 2.06 (P = 0.04)								
**Systemic chemotherapy**								
Zhang, Z.2016	1.26	0.6	60	1.02	0.75	60	7.0%	0.35 [-0.01, 0.71]
Yuan,J.2011	1.91	0.63	34	1.47	0.46	34	6.7%	0.79 [0.29, 1.28]
**Subtotal (95% CI)**			**94**			**94**	**13.7%**	**0.54 [0.11, 0.96]**
Heterogeneity: Tau² = 0.05; Chi² = 1.96, df = 1 (P = 0.16); I² = 49%								
Test for overall effect: Z = 2.48 (P = 0.01)								
**Total (95% CI)**			**531**			**537**	**100.0%**	**0.74 [0.30, 1.19]**
Heterogeneity: Tau² = 0.71; Chi² = 165.67, df = 14 (P < 0.00001); I² = 92%								
Test for overall effect: Z = 3.27 (P = 0.001)								
Test for subgroup differences: Chi² = 26.52, df = 4 (P < 0.0001), I² = 84.9%								

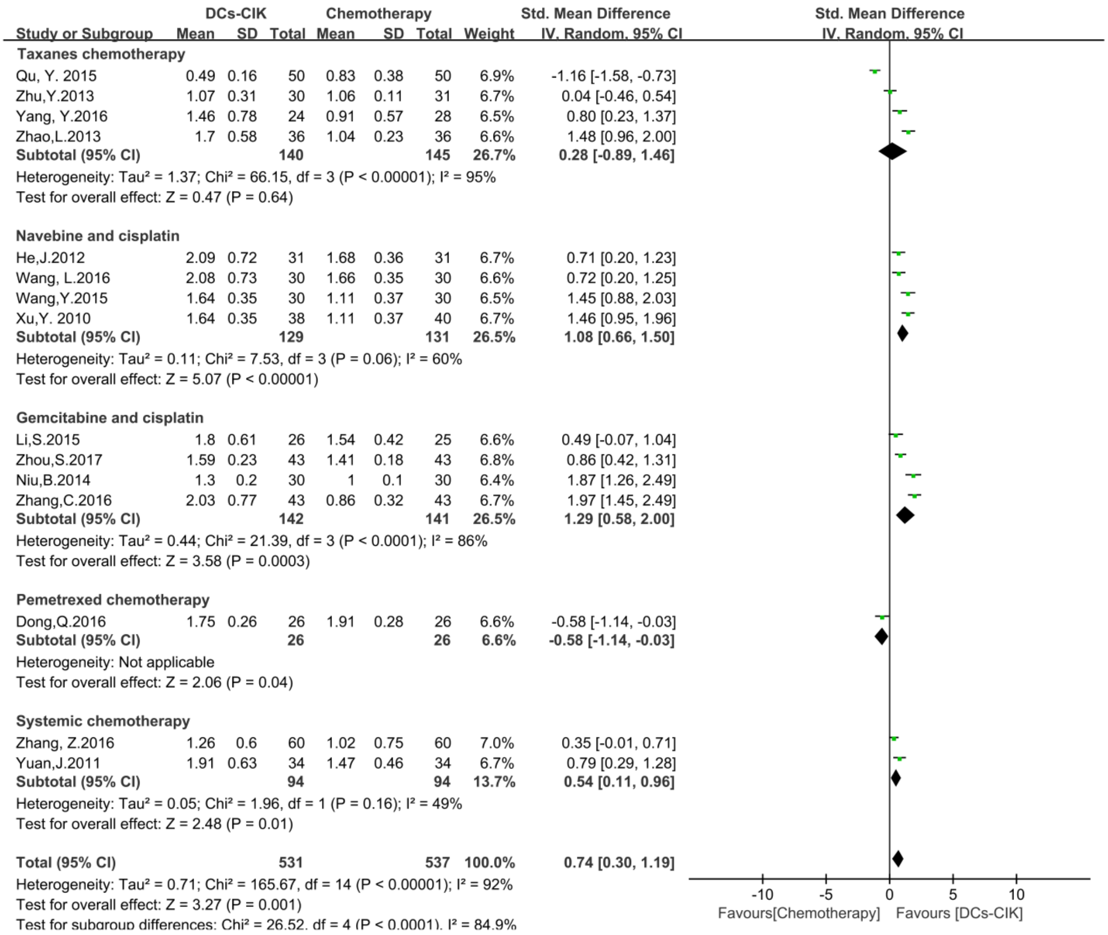

Figure S12.Subgroups analysis of $CD4^+$/$CD8^+$ T cell via combinations with chemotherapy
